# Targeting a Leukemia‐Relevant Protein–Protein Interface: N‐Aryl Anthranilic Acids as Disruptors of the c‐Myb–CBP/p300 KIX Interaction

**DOI:** 10.1111/cbdd.70415

**Published:** 2026-10-07

**Authors:** Haifa E. Alfassam, Emadeldin M. Kamel, Sarah I. Othman, Hanan M. Alharbi, Hassan A. Rudayni, Ahmed A. Allam, Al Mokhtar Lamsabhi

**Affiliations:** ^1^ Department of Biology, College of Science Princess Nourah bint Abdulrahman University Riyadh Saudi Arabia; ^2^ Chemistry Department, Faculty of Science Beni‐Suef University Beni‐Suef Egypt; ^3^ Department of Biology, College of Science Imam Mohammad Ibn Saud Islamic University (IMSIU) Riyadh Saudi Arabia; ^4^ Departamento de Química and Institute for Advanced Research in Chemical Science (IAdChem), Facultad de Ciencias Módulo 13, Universidad Autónoma de Madrid Madrid Spain

**Keywords:** CBP/p300 KIX, c‐Myb, microscale thermophoresis, N‐aryl anthranilic acids, protein–protein interaction

## Abstract

The interaction between c‐Myb and the CBP/p300 KIX domain is a key transcriptional event in acute myeloid leukemia (AML) and represents an attractive target for small‐molecule intervention. In this study, we applied an integrated computational–experimental strategy to identify new N‐aryl anthranilic acid disruptors of the c‐Myb–KIX protein–protein interaction. A scaffold‐focused library of 3656 N‐aryl anthranilic acid derivatives was filtered, docked to the c‐Myb‐binding groove of KIX (PDB 2AGH), and the top hits were further evaluated by MD simulations, MM/PBSA, FEL, and ADMET analyses. This workflow prioritized four hits (C1–C4) for experimental testing. Computational analyses showed that all four compounds remained bound within the α1/α3 Myb‐facing groove, but differed markedly in their predicted effects on the c‐Myb–KIX interface. Among them, C3 emerged as the strongest predicted disruptor, whereas C1 was predicted to stabilize the complex. The $\Delta \Delta G_{\mathrm{PPI}}$ analysis further showed that C3 produced the largest destabilizing effect on the c‐Myb–KIX interface (+4.57 kcal/mol), followed by C2 (+2.30 kcal/mol) and C4 (+1.40 kcal/mol), whereas C1 stabilized the complex (−6.47 kcal/mol). These predictions were validated by microscale thermophoresis (MST). The binary c‐Myb–KIX interaction showed an apparent *K*_*d*_ of 26.71 ± 0.54 μM, and the compounds inhibited complex formation with IC_50_ values of 99.6 ± 20.5 μM (C1), 54.7 ± 22.1 μM (C2), 16.5 ± 0.8 μM (C3), and 27.2 ± 2.7 μM (C4). Notably, C3 outperformed the reference inhibitor Naphthol AS‐E phosphate (28.8 ± 3.1 μM). Overall, these findings identify C3 as a promising lead and establish N‐aryl anthranilic acids as a tractable scaffold for targeting the c‐Myb–CBP/p300 KIX interaction.

## Graphical abstract

An integrated virtual screening, MD/MM‐PBSA, and MST workflow identifies N‐aryl anthranilic acids as disruptors of the leukemia‐relevant c‐Myb–CBP/p300 KIX interface. Hit C3 destabilizes the complex and inhibits c‐Myb–KIX binding more potently than Naphthol AS‐E phosphate.

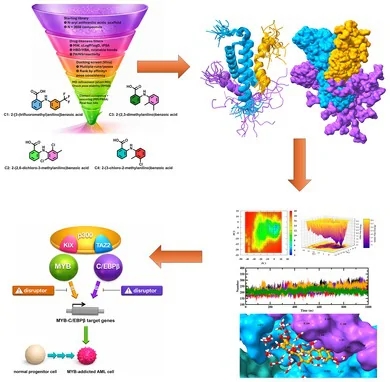

## 1 Introduction

The transcription factor c‐Myb is a central regulator of hematopoietic gene expression and is expressed predominantly in hematopoietic progenitor cells, where it is required for normal proliferation and differentiation programs (Thakur et al. 2014). A major determinant of c‐Myb transactivation is its recruitment of the paralogous coactivators CBP and p300, whose KIX domain recognizes the c‐Myb transactivation region and helps assemble productive transcriptional complexes (Ledsaak et al. 2016). In acute myeloid leukemia (AML), this axis is therapeutically attractive because AML cells are unusually dependent on high MYB activity, and genetic evidence has shown that disruption of the c‐Myb–p300 interaction impairs leukemogenic transformation driven by major AML oncogenes (Pattabiraman et al. 2014; Schmidt and Klempnauer 2022; Yusenko et al. 2021).

Structurally, the KIX domain is a highly conserved, independently folded three‐helix bundle that functions as a docking platform for multiple transcriptional activators (Zor et al. 2004). Early structural studies showed that the c‐Myb transactivation domain binds KIX as a bent amphipathic helix within the hydrophobic groove formed mainly by the α1 and α3 helices, with Leu302 of c‐Myb making a dominant contribution to binding (De Guzman et al. 2006). This framework was extended by the ternary NMR structure deposited as PDB 2AGH, which captures c‐Myb and MLL bound simultaneously to opposite faces of the CBP KIX domain. In that ternary complex, MLL binding stabilizes regions of KIX and provides a structural basis for cooperative enhancement of c‐Myb recognition. Accordingly, Figure 1A,B offers a useful structural rationale for targeting the c‐Myb‐facing groove as a defined protein–protein interaction surface within the broader MYB transcriptional machinery (De Guzman et al. 2006; Thakur et al. 2014; Zor et al. 2004).

**FIGURE 1:**
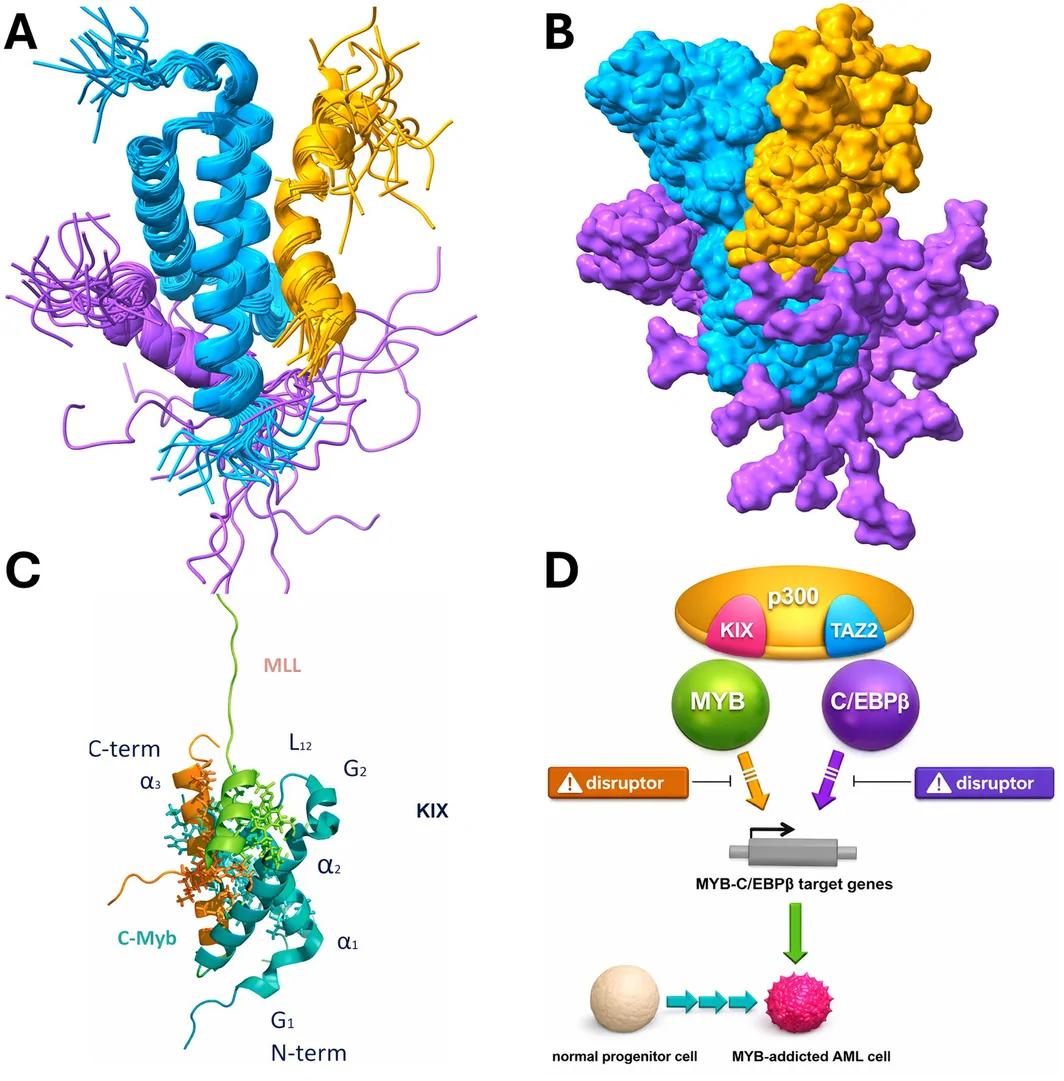
Structural basis and functional context of the c‐Myb–KIX interaction. (A) Superposition of the deposited NMR ensemble for the ternary CBP KIX:c‐Myb:MLL complex (PDB 2AGH) (R. N. De Guzman et al. 2006). The KIX domain (residues 586–672) is shown in teal, the c‐Myb transactivation domain (residues 291–310) in slate blue, and the MLL transactivation domain (residues 840–869) in gold. (B) Surface representation of the same complex, colored as in panel A, illustrating the c‐Myb‐binding groove located at the α1/α3 interface of KIX, which was used as the docking site in the present study, and the distinct α2/α3 site occupied by MLL. (C) Ribbon view of a representative conformer highlighting the characteristic KIX fold, which comprises three α‐helices (α1–α3) capped by two short 3_10_ helices (G₁ and G_2_). Residues shaping the approximately 200 Å^3^ hydrophobic pocket—Leu603, Tyr650, Ala654, Ile657, and Tyr658 of KIX—together with Leu302 of c‐Myb, are shown as sticks (Schmidt and Klempnauer 2022). (D) Schematic model of the MYB/C/EBPβ transcriptional module, in which p300 coordinates MYB through KIX and C/EBPβ through TAZ2; disruption of these coactivator‐dependent interactions is expected to attenuate MYB/C/EBPβ target‐gene expression and suppress the MYB‐dependent leukemic state.

Although transcription factor–coactivator interfaces are often considered difficult to drug, the c‐Myb–KIX interaction has already yielded meaningful proof of concept for pharmacologic intervention (Uttarkar et al. 2015). Naphthol AS‐E phosphate was reported as the first low‐molecular‐weight compound to inhibit Myb activity by blocking its interaction with the KIX domain of p300, and later work showed that additional chemical matter and peptide‐based antagonists can suppress MYB‐dependent leukemia phenotypes (Ramaswamy et al. 2018; Uttarkar et al. 2015; Uttarkar et al. 2016). In particular, the peptidomimetic MYBMIM and the high‐affinity dual‐site ligand MybLL‐tide demonstrated that KIX‐directed antagonism can modulate MYB‐driven transcription in AML models (Ramaswamy et al. 2018). Taken together, these studies support the tractability of the target while also underscoring the need for additional, chemically distinct small‐molecule scaffolds capable of engaging the c‐Myb/KIX interface (Joy et al. 2021).

Small‐molecule targeting of protein–protein interactions remains challenging because these interfaces commonly extend over approximately 1500–3000 Å^2^ and are often comparatively flat, solvent‐exposed, and conformationally dynamic (Kamel et al. 2026, 2026). Their binding energetics, however, are not distributed uniformly across the entire interface but are frequently concentrated within localized hot spots that make disproportionately large contributions to complex formation. Small molecules therefore need not reproduce or cover the complete protein‐contact surface; instead, they may inhibit a PPI by occupying a compact hot‐spot cluster or an adjacent ligandable pocket (Arkin et al. 2014; Smith and Gestwicki 2012). In the c‐Myb–KIX complex, such an opportunity is provided by the localized hydrophobic depression at the α1/α3 surface of KIX, which is lined principally by Leu603, Tyr650, Ala654, Ile657, and Tyr658 and accommodates the functionally important Leu302 side chain of c‐Myb (De Guzman et al. 2006; Zor et al. 2004). The approximately 200 Å^3^ value assigned to this feature describes the volume of this local subpocket and not the total area of the broader c‐Myb–KIX interface. Accordingly, the present strategy was designed to target this hot‐spot‐containing groove rather than to cover the entire PPI surface. Although its shallow and flexible nature may limit small‐molecule affinity and selectivity, the presence of a pre‐existing hydrophobic pocket, together with previous chemical and peptidomimetic disruption of the c‐Myb–KIX interaction, supports its tractability for structure‐guided ligand discovery.

Against this background, we sought to identify new small‐molecule disruptors of the c‐Myb–CBP/p300 KIX interaction from an N‐aryl anthranilic acid scaffold library. Using the structural context provided by the KIX:c‐Myb:MLL complex, we first carried out virtual screening to prioritize candidate ligands for the c‐Myb‐binding surface. Selected hits were then advanced to 500 ns molecular dynamics simulations to evaluate binding stability and ligand‐induced conformational behavior in the KIX binding pocket. Finally, the computational predictions were tested experimentally by microscale thermophoresis, providing solution‐phase validation of lead compounds able to perturb this therapeutically relevant transcriptional interface.

## 2 Experimental

### 2.1 Virtual Screening

A structure‐guided virtual screening workflow was used to identify N‐aryl anthranilic acid derivatives with the potential to disrupt the c‐Myb‐binding surface of the KIX domain. The receptor model was based on the NMR structure of the ternary CBP KIX:c‐Myb:MLL complex (PDB 2AGH), in which c‐Myb and MLL occupy two distinct binding sites on KIX (R. N. De Guzman et al. 2006). The initial library comprised 3656 N‐aryl anthranilic acid analogues (Supporting Information), which were first filtered using standard drug‐likeness criteria, including molecular weight, cLogP/logD, topological polar surface area, hydrogen‐bond donor/acceptor counts, and the number of rotatable bonds; compounds containing PAINS motifs or obviously reactive substructures were excluded at this stage (Baell and Holloway 2010). The filtered set was then docked into the Myb‐facing groove of KIX using AutoDock Vina, and multiple independent docking runs were carried out for each ligand to sample alternative binding poses and improve confidence in pose reproducibility (Trott and Olson 2010). Hits were prioritized by combining predicted binding affinity with pose consistency across runs, after which the top‐ranked complexes were subjected to short molecular dynamics refinement to assess whether the docked orientation remained stable, using ligand/protein RMSD as the primary stability criterion. Surviving candidates were then compared by contact‐occupancy analysis and MM/PBSA rescoring, and this funnel‐based procedure yielded four final compounds (C1–C4) for subsequent long‐timescale MD simulations and experimental validation (Genheden and Ryde 2015).

### 2.2 Molecular Docking

#### 2.2.1 Ligand Preparation

All N‐aryl anthranilic acid derivatives included in the virtual‐screening campaign were prepared using a uniform quantum‐chemical protocol (Alqhtani et al. 2025; Alwaili et al. 2025). For each molecule, an initial 3D model was generated and then energy‐minimized by density functional theory (DFT) in Gaussian 16 (Revision C.01) using the B3LYP/6‐31G(d, p) level of theory (Frisch et al. 2016; Lee et al. 1988). To verify that the optimized structures represented true minima on the potential‐energy surface, frequency calculations were performed and only geometries with no imaginary vibrational frequencies were retained. The optimized ligands were subsequently processed in AutoDockTools, where polar hydrogens were added, Gasteiger charges were assigned, and the rotatable bonds required for flexible docking were defined (Morris et al. 2009). The final ligand files were exported in PDBQT format for use in AutoDock Vina (Trott and Olson 2010).

#### 2.2.2 Receptor Preparation

Docking was carried out using the KIX domain of CBP extracted from the solution NMR structure of the ternary KIX:c‐Myb:MLL complex (PDB ID: 2AGH) (De Guzman et al. 2006). The protein structure was prepared in AutoDockTools by removing unnecessary solvent records, adding polar hydrogens, and assigning the atomic charges needed for Vina calculations. The search space was defined around the c‐Myb‐binding groove located at the α1/α3 interface of KIX, hereafter referred to as the Myb‐facing groove. This site was identified directly from the experimental 2AGH structure as the surface occupied by the c‐Myb transactivation domain and is illustrated in Figure 1B,C (De Guzman et al. 2006). The distinct MLL‐binding site, positioned on the opposite face of KIX, was not used as the primary docking target. A grid box of 40 Å × 40 Å × 52 Å was centered at *x* = −4.452, *y* = 13.832, *z* = 11.828, allowing complete coverage of the Myb‐facing groove and its surrounding interfacial pocket.

Although the CBP KIX domain in PDB 2AGH originates from *Mus musculus* , its suitability as a structural model of the human target was verified by sequence comparison. The complete 87‐residue KIX sequence represented in 2AGH is identical to the corresponding human CREBBP KIX sequence, as confirmed by comparison of the reviewed mouse and human CREBBP sequences (UniProt accession numbers P45481 and Q92793, respectively). Consequently, all residues forming the targeted α1/α3 c‐Myb‐binding groove are conserved, and no species‐dependent substitution occurs within the receptor surface used for docking. PDB 2AGH was therefore retained because it provides an experimentally determined ternary c‐Myb–KIX–MLL structure that captures the cooperative KIX conformation relevant to c‐Myb recognition (De Guzman et al. 2006). The translational relevance of this model was further supported by experimental validation using the human eGFP/hc‐Myb(241–390) and KIX‐containing p300(1–672) constructs in the MST assay (Uttarkar et al. 2017).

#### 2.2.3 Molecular Docking

Docking calculations were performed with AutoDock Vina v1.2.5 using the prepared KIX receptor and the grid centered on the α1/α3 c‐Myb recognition site (Trott and Olson 2010). During docking, the receptor was treated as rigid, whereas each ligand was allowed full torsional flexibility. To improve conformational sampling and increase the likelihood of identifying reproducible binding modes, the exhaustiveness parameter was set to 16. Each ligand was docked in three independent runs, and up to 20 poses per run were retained within an energy range of 3 kcal/mol. Independent random seeds were used for the separate runs to assess the reproducibility of the predicted binding orientations. After completion of docking, all poses obtained for a given compound were pooled and grouped according to structural similarity using a 2.0 Å heavy‐atom RMSD cutoff, with clustering referenced to the top‐scoring pose. The resulting complexes were then visually examined in PyMOL 2.6. Binding modes were prioritized when they were positioned clearly within the intended KIX groove, reproduced chemically plausible hydrophobic and aromatic contacts expected for occupation of the c‐Myb site, and showed at least one reasonable polar interaction, such as hydrogen bonding or electrostatic complementarity. For compounds selected for the subsequent MD studies, the starting complex was chosen from the lowest‐energy pose within the most relevant cluster that also satisfied these interaction criteria. When multiple clusters met the selection requirements, preference was given to the pose that displayed the most reproducible placement in the α1/α3 groove across the three independent docking runs.

#### 2.2.4 Docking Validation

The docking protocol was validated by assessing whether the adopted search space and scoring procedure reproducibly recovered binding modes within the experimentally defined c‐Myb recognition groove of KIX. The benchmark region was taken from the KIX:c‐Myb:MLL NMR structure (PDB 2AGH), in which the c‐Myb transactivation domain occupies the α1/α3 interface of KIX. Accordingly, the docking setup was considered appropriate only when independent docking runs consistently placed the ligands in this groove, rather than in the distal MLL‐binding site, and generated orientations compatible with the known topology of the c‐Myb interface. For each compound, poses obtained from the three independent AutoDock Vina runs were pooled and clustered using a 2.0 Å heavy‐atom RMSD cutoff to evaluate pose convergence and run‐to‐run reproducibility. The protocol was judged reliable when the dominant low‐energy cluster was repeatedly recovered across runs and positioned the ligand within the hydrophobic pocket defined by key KIX residues Leu603, Tyr650, Ala654, Ile657, and Tyr658 in a manner consistent with occupation of the native Myb groove. Additional visual inspection in PyMOL 2.6 was used to remove geometrically implausible poses and to confirm reasonable hydrophobic, aromatic, and polar contacts. On this basis, confidence in the docking results was assigned according to site recovery, pose reproducibility, and chemical plausibility of the predicted KIX–ligand complexes.

### 2.3 Molecular Dynamics Simulations

To assess the stability of the docked binding modes, 500 ns molecular dynamics (MD) simulations were performed for the selected KIX–ligand complexes using GROMACS 2022.4 (Lindahl et al. 2001; Van Der Spoel et al. 2005). The CBP KIX domain was described with the CHARMM36m force field, and explicit solvent was represented using the CHARMM‐compatible TIP3P water model (Huang et al. 2017). Parameters for the N‐aryl anthranilic acid ligands were generated through the CGenFF workflow and converted into GROMACS‐compatible topology files to maintain consistency with the protein force field (Vanommeslaeghe and MacKerell Jr. 2012). Each complex was placed in a periodic dodecahedral box, solvated with water molecules, and neutralized by adding the appropriate counterions. Before production dynamics, the systems were subjected to steepest‐descent energy minimization to remove unfavorable contacts, followed by sequential equilibration under the NVT and NPT ensembles. The production simulations were run with a 2 fs time step at 300 K, controlled with the velocity‐rescaling thermostat, while the pressure was maintained at 1 bar using the Parrinello–Rahman barostat (Bussi et al. 2007). Short‐range van der Waals and Coulomb interactions were calculated using a 1.2 nm cutoff, and long‐range electrostatic interactions were treated with the Particle Mesh Ewald (PME) method. All bonds involving hydrogen atoms were constrained using the LINCS algorithm, enabling stable long‐timescale integration under periodic boundary conditions (Hess et al. 1997). After equilibration, each system was simulated for 500 ns, and the resulting trajectories were evaluated using standard thermodynamic and structural descriptors, including temperature, pressure, density, potential energy, and backbone RMSD, to confirm equilibration before downstream analysis.

### 2.4 MM/PBSA Free‐Energy Calculations

To further evaluate the behavior of the selected N‐aryl anthranilic acid derivatives after docking and MD simulation, MM/PBSA calculations were carried out on equilibrated structures extracted from the MD trajectories using gmx_MMPBSA v1.6.3 (Valdés‐Tresanco et al. 2021). Two related questions were addressed in this analysis: first, whether ligand binding altered the energetic stability of the c‐Myb–KIX protein–protein interface; and second, how strongly each ligand associated with the KIX‐containing target complex. For each simulated system, frames were collected from the last 100 ns of the 500 ns trajectory (400–500 ns), and the reported values are presented as mean ± standard deviation (SD) over the sampled structures.

In all calculations, binding energies were estimated according to the standard MM/PBSA formalism:

$$\Delta G_{\text{bind}}=\Delta E_{\mathrm{MM}}+\Delta G_{\text{solv}}-\mathrm{T\Delta S}$$

where $\Delta E_{\mathrm{MM}}$ corresponds to the molecular‐mechanics term, dominated mainly by van der Waals and electrostatic interactions, and $\Delta G_{\text{solv}}$ represents the solvation contribution. The polar solvation energy was obtained by solving the Poisson‐Boltzmann equation, whereas the nonpolar solvation term was estimated from the solvent‐accessible surface area (SASA). Unless otherwise stated, the entropic contribution $\left(T\Delta S\right)$ was not included. Consequently, the MM/PBSA values reported here were treated as relative energetic measures useful for comparing compounds and identifying trends, rather than as absolute thermodynamic free energies.

#### 2.4.1 Ligand‐Induced Modulation of the c‐Myb–KIX Interaction $\left(\Delta \Delta G_{\mathrm{PPI}}\right)$

To examine whether ligand binding weakened or reinforced the c‐Myb–KIX association, a differential MM/PBSA approach was applied in which the interaction energy of the ligand‐bound (holo) system was compared with that of the corresponding ligand‐free (apo) reference:

$$\Delta \Delta G_{\mathrm{PPI}}=\Delta G_{\mathrm{PPI}}^{\text{holo}}-\Delta G_{\mathrm{PPI}}^{\mathrm{apo}}$$

In practical terms, this was defined as:

$$\Delta \Delta G_{\mathrm{PPI}}=\Delta G_{\text{bind}}\left(c-\mathrm{Myb}–\mathrm{KIX},\text{with ligand}\right)-\Delta G_{\text{bind}}\left(c-\mathrm{Myb}–\mathrm{KIX},\mathrm{apo}\right)$$

The holo term was derived from snapshots taken from the ligand‐containing trajectories, while the apo reference was obtained from the corresponding ligand‐free system evaluated under the same calculation protocol. This strategy isolates the energetic effect of ligand binding on the c‐Myb–KIX interface. Under this definition, a positive $\Delta \Delta G_{\mathrm{PPI}}$ value indicates weakening of the protein–protein interaction, consistent with ligand‐induced destabilization, whereas a negative value suggests stabilization relative to the apo state.

#### 2.4.2 MM/PBSA Estimation of Ligand Binding to the Target Complex

An additional MM/PBSA analysis was performed to estimate the relative binding strength of each ligand toward the target complex. For every ligand‐bound trajectory, the protein–ligand interaction energy was calculated as:

$$\Delta G_{\text{bind}}^{\text{protein}-\text{ligand}}=G_{\text{complex}}-\left(G_{\text{protein}}+G_{\text{ligand}}\right)$$

The resulting values from the selected 400–500 ns interval were averaged to provide a trajectory‐based estimate of ligand affinity. These MM/PBSA‐derived energies were interpreted together with docking scores, MD stability metrics, and the calculated $\Delta \Delta G_{\mathrm{PPI}}$ values to support compound prioritization. During final hit selection, energetic results were considered alongside structural and dynamic descriptors extracted from the MD simulations, including RMSD behavior, persistence of intermolecular contacts, and interaction occupancy, so that compounds were prioritized not only for favorable binding to KIX, but also for their predicted ability to interfere with the c‐Myb–KIX interaction.

### 2.5 Free Energy Landscape (FEL) Analysis

The conformational space explored by the KIX–ligand complexes during the 500 ns MD simulations was characterized by principal component analysis (PCA) using the covariance‐analysis tools available in GROMACS. Before PCA, the trajectories were preprocessed to remove translational artifacts and to center/fit the structures to a reference frame. A covariance matrix describing the positional fluctuations of the selected atoms was then generated and diagonalized, yielding eigenvectors, which define the dominant concerted motions, and eigenvalues, which quantify the amplitude of those motions. The MD trajectories were subsequently projected onto the resulting principal axes, and the first two components (PC1 and PC2) were retained to represent the major large‐scale conformational changes sampled during the simulations. These two coordinates were then used to map the accessible conformational space in two dimensions.

Using the PC1–PC2 projections as reaction coordinates, the FEL was reconstructed in GROMACS with the SHAM procedure, which estimates Gibbs free‐energy surfaces by Boltzmann inversion of multidimensional histograms derived from the conformational population distribution. The free energy of each region was calculated according to:

$$G\left(\mathrm{PC}1,\mathrm{PC}2\right)=-k_{B}\mathrm{Tln}P\left(\mathrm{PC}1,\mathrm{PC}2\right)$$

up to an arbitrary constant, where $k_{B}$ is the Boltzmann constant, T is the absolute temperature, and $P\left(\mathrm{PC}1,\mathrm{PC}2\right)$ is the probability density associated with a given conformational state in the projected space. The resulting two‐dimensional FEL maps were initially written in XPM format and were then converted into tabulated text files using a custom Python script to facilitate visualization and quantitative examination of low‐energy minima and the transition regions connecting them.

### 2.6 In Silico ADMET Evaluation

The selected N‐aryl anthranilic acid derivatives were further evaluated for their pharmacokinetic and safety profiles using the ADMETlab 3.0 web server (Fu et al. 2024). ADMETlab 3.0 is an online platform for the prediction of physicochemical, medicinal chemistry, absorption, distribution, metabolism, excretion, and toxicity properties, and it provides broad endpoint coverage together with model‐based confidence support. For each compound, the molecular structure was submitted to the server and the predicted ADMET‐related parameters were retrieved and compared to support compound prioritization in the later stages of the study. The resulting profiles were used as a complementary filter to identify compounds with more favorable drug‐likeness and developability characteristics.

### 2.7 Microscale Thermophoresis Assay

#### 2.7.1 Chemicals, Reagents, and Proteins

Microscale thermophoresis experiments were carried out with the four N‐aryl anthranilic acid derivatives prioritized by the in silico workflow: C1 [2‐(3‐trifluoromethylanilino)benzoic acid], C2 [2‐(2,6‐dichloro‐3‐methylanilino)benzoic acid], C3 [2‐(2,3‐dimethylanilino)benzoic acid], and C4 [2‐(3‐chloro‐2‐methylanilino)benzoic acid]. Naphthol AS‐E phosphate (KG‐501) was included as a reference inhibitor. Studied compounds were obtained from MedChemExpress. The assay format followed the previously reported Myb–KIX MST system, in which eGFP/hc‐Myb(241–390) was used as the fluorescent tracer and soluble p300(1–672) served as the KIX‐containing binding partner (Uttarkar et al. 2015). Recombinant proteins were produced and purified according to the established protocol described for this assay (Uttarkar et al. 2015).

#### 2.7.2 Verification of the Binary Interaction

Before inhibitor testing, the binding of eGFP/hc‐Myb(241–390) to p300(1–672) was confirmed under the final experimental conditions. The fluorescent Myb construct was kept at 50 nM, whereas p300(1–672) was titrated over a range of 0.85–28 μM. The normalized fluorescence signal was then plotted against p300 concentration, and the resulting binding isotherm was used to determine the apparent K_d_ for the Myb–KIX interaction (Uttarkar et al. 2015). This control experiment was performed in three independent replicates to verify assay robustness.

#### 2.7.3 Compound Dilution and Sample Assembly

For inhibition measurements, eGFP/hc‐Myb(241–390) and p300(1–672) were used at final concentrations of 50–28 μM, respectively. Each test compound was prepared as a serial dilution spanning 50–1.52 nM. Stock solutions were first made in DMSO and then diluted with assay buffer so that every sample, including the vehicle controls, contained the same final proportion of solvent. Equal volumes of the compound dilution series and a 2× protein master mix were combined to generate the assay samples. Buffer composition and instrumental settings were kept identical to those reported for the original Myb–KIX MST assay.

#### 2.7.4 MST Acquisition

After sample preparation, mixtures were vortexed gently, briefly centrifuged, and loaded into MST capillaries. Capillary quality was checked before measurement, and samples showing adsorption to the capillary wall or atypical fluorescence behavior were excluded from analysis. Thermophoresis recordings were then collected and processed according to the previously established assay workflow. Under these conditions, disruption of the Myb–KIX complex by an active compound was detected as an increase in normalized fluorescence (*F*_norm_), consistent with displacement of the bound state.

#### 2.7.5 Control Conditions

Each experiment included a vehicle control containing both protein partners together with the same final concentration of DMSO, but without inhibitor, to define the fully bound reference state. An additional sample containing eGFP/hc‐Myb(241–390) in the absence of p300(1–672) was used to represent the unbound condition. To detect compound‐related optical artifacts, every test molecule was also evaluated in a control containing the fluorescent Myb construct plus compound, but lacking p300(1–672), thereby enabling identification of autofluorescence, quenching, or signal enhancement unrelated to inhibition of the protein–protein interaction. KG‐501 was included in each run as a positive‐control disruptor. Initial fluorescence values were reviewed across the full concentration range, and any dataset showing substantial baseline drift or abnormal traces was discarded and repeated.

#### 2.7.6 Data Analysis

For each compound, normalized fluorescence was plotted as a function of the logarithm of inhibitor concentration, and the data were fitted by nonlinear regression using a variable‐slope sigmoidal inhibition model to obtain IC_50_ values. Every compound was tested in three independent experiments, and the final results are reported as mean ± SD. The apparent K_d_ obtained from the direct‐binding experiment is presented separately as a quality‐control parameter for the assay, whereas IC_50_ values were determined for C1–C4 in the inhibition format. The response obtained with KG‐501 was used to confirm that the assay remained sensitive to pharmacologic disruption of the Myb–KIX interaction.

## 3 Results and Discussion

### 3.1 A Multi‐Parameter Virtual‐Screening Funnel Efficiently Reduced 3656 N‐Aryl Anthranilic Acids to Four Prioritized Candidates for KIX Targeting

As shown in Figure 2, the virtual‐screening campaign applied a sequential filtering strategy that progressively narrowed a scaffold‐focused library of 3656 N‐aryl anthranilic acids to four final candidates (C1–C4) predicted to target the c‐Myb‐binding groove of the KIX domain. The workflow began with physicochemical and medicinal‐chemistry filtering based on molecular weight, cLogP/logD, topological polar surface area, hydrogen‐bond donor/acceptor counts, rotatable bonds, and PAINS/reactivity alerts, thereby removing compounds less compatible with drug‐like behavior and reducing the risk of false‐positive hits. The remaining molecules were then evaluated by AutoDock Vina against the α1/α3 Myb‐facing groove, where compounds were ranked not only by docking score but also by pose consistency across multiple runs, an important criterion for a shallow protein–protein interaction surface such as KIX. Top‐ranked ligands were subsequently subjected to short MD refinement, and only those maintaining acceptable pose stability by RMSD were advanced. Final selection was based on the combined assessment of contact occupancy and MM/PBSA rescoring, which helped distinguish compounds showing persistent and energetically favorable engagement of the target pocket from those supported only by initial docking. This stepwise process ultimately yielded four structurally related but substitutionally distinct hits—C1, C2, C3, and C4—that were considered the most credible candidates for further 500 ns MD simulations, energetic analysis, and experimental validation by microscale thermophoresis.

**FIGURE 2:**
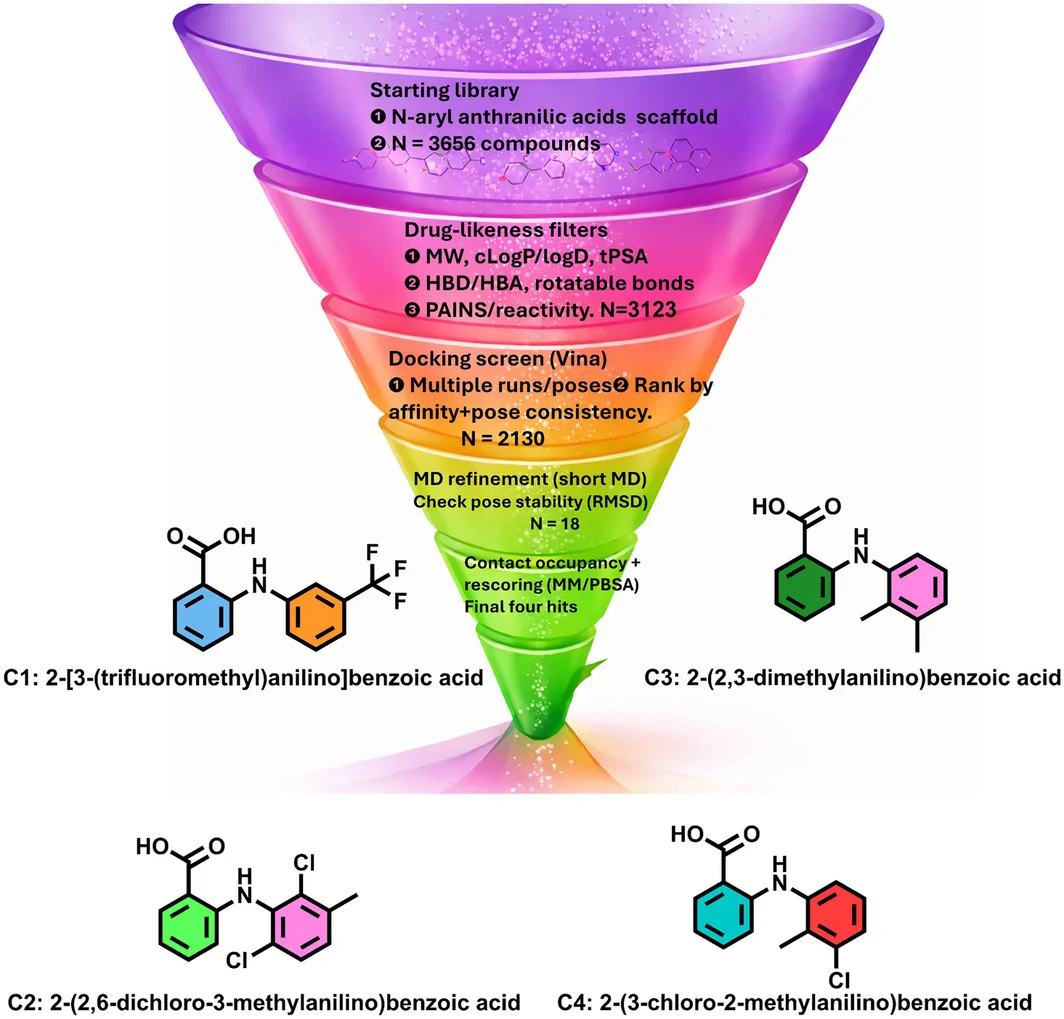
Virtual‐screening workflow used to identify N‐aryl anthranilic acid hits targeting the c‐Myb‐binding groove of KIX.

### 3.2 Docking Predicts That C1–C4 Occupy the c‐Myb‐Facing KIX Groove and Reproduce Key Interaction Features of the Reference Inhibitor

The docking results shown in Figure 3 indicate that the four prioritized N‐aryl anthranilic acid derivatives are predicted to bind within the same α1/α3 c‐Myb‐recognition groove of KIX that accommodates the reference inhibitor Naphthol AS‐E phosphate. In panel A, all four hits adopt poses centered in the interfacial cavity rather than the distal surface, while panel C shows that their surface placement remains confined to the intended binding region. This common localization is important because it suggests that the virtual‐screening funnel did not merely identify compounds with favorable docking scores, but rather enriched for ligands that converged on the biologically relevant Myb‐facing pocket. Relative to Naphthol AS‐E phosphate, which displays a broader interaction network driven by its highly polar phosphate group, the anthranilic acid hits occupy the same general site with a simpler binding pattern in which the carboxylate‐bearing anthranilic acid headgroup is positioned toward the polar rim of the groove and the substituted aniline ring extends into the more hydrophobic portion of the pocket.

**FIGURE 3:**
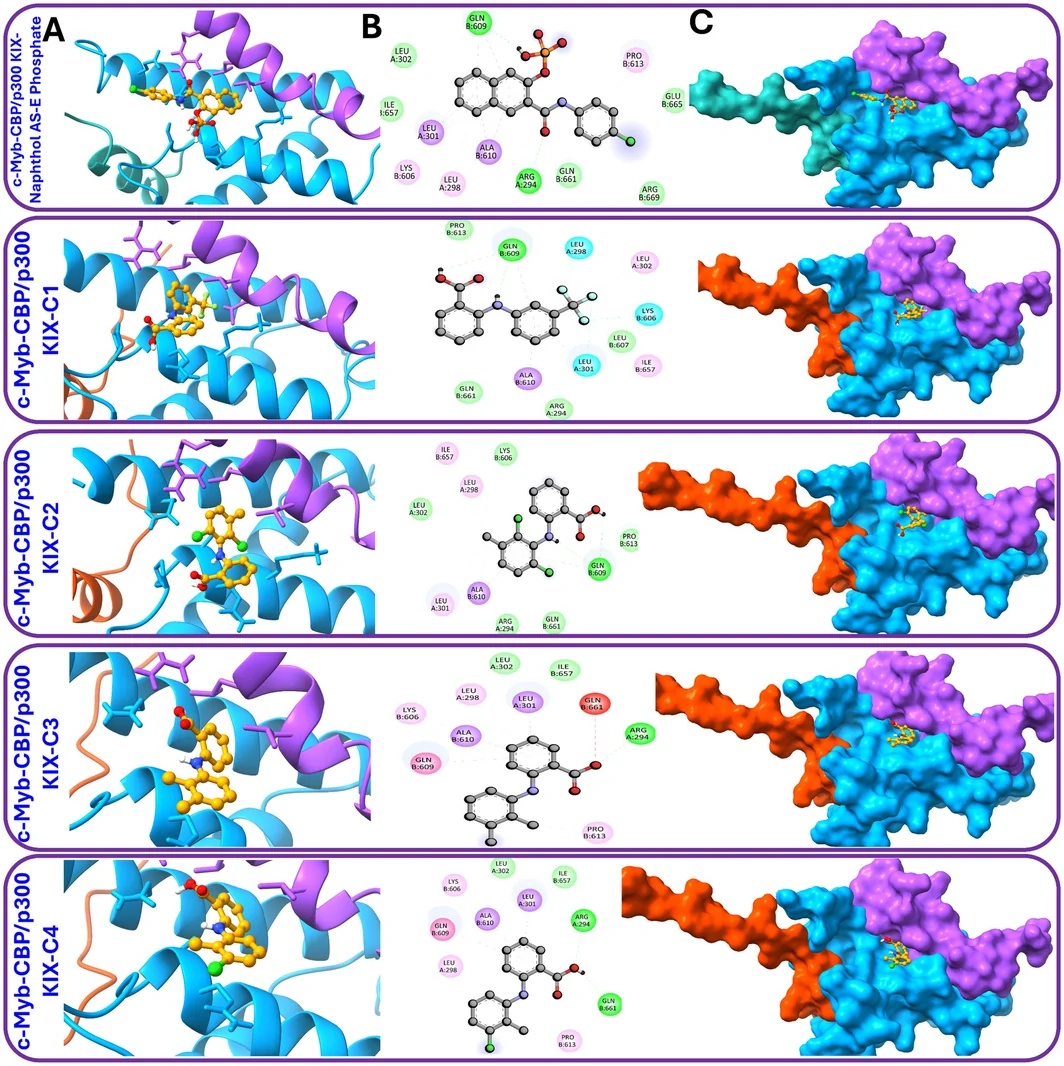
Docking poses and interaction profiles of the reference inhibitor and the four selected N‐aryl anthranilic acid hits in the c‐Myb‐facing groove of KIX. Representative docking results are shown for Naphthol AS‐E phosphate (reference inhibitor) and the four prioritized compounds C1–C4. For each ligand, (A) shows the predicted binding pose in the c‐Myb‐binding groove of the CBP/p300 KIX domain in cartoon representation, (B) presents the corresponding 2D ligand–residue interaction map, and (C) displays the docked complex in surface view to illustrate ligand placement within the interfacial pocket. In all cases, the ligands are predicted to occupy the α1/α3 Myb‐recognition groove rather than the distal MLL‐binding surface, supporting a common binding mode for the hit series.

The 2D interaction maps in panel B further support a shared recognition mode across the hit series. Despite differences in substitution pattern, all four compounds retain contacts with residues that define the docked KIX pocket, most consistently including Arg294, Leu301/Leu302, Ala610, Gln609, Pro613, and Ile/Leu657, with additional contributions from Gln661 and neighboring residues in several poses. This pattern indicates that ligand recognition is governed by a combination of hydrophobic packing and limited polar anchoring, rather than by an extensive hydrogen‐bonding network. In this context, the aromatic scaffold appears well suited for engagement of the shallow KIX groove, while the acidic group likely contributes positional stabilization through electrostatic or hydrogen‐bond‐compatible interactions at the pocket entrance. Notably, the docked pose of Naphthol AS‐E phosphate follows the same overall topography but shows more pronounced polar complementarity, which is consistent with its use as a reference disruptor and provides a useful benchmark for evaluating whether the new scaffold can mimic its site occupancy without reproducing its exact chemistry.

Comparison of the four hits suggests that substituent‐dependent hydrophobic complementarity is likely to influence binding quality within the groove. The halogenated analogues C1, C2, and C4 appear to place their terminal aryl groups deeply within the lipophilic region of the pocket, where fluorine‐ or chlorine‐containing substituents may strengthen van der Waals contacts and improve shape matching. In contrast, C3, which bears only methyl substituents, still adopts the same binding region but appears to rely more on aromatic packing than on the additional lipophilic reinforcement provided by halogens. Among the series, C1 and C2 appear particularly well aligned with the reference pocket geometry, whereas C4 preserves the same general binding mode with a slightly more compact interaction pattern. C3 remains compatible with groove occupation but may represent a somewhat less extensively anchored pose. Overall, the docking analysis supports the conclusion that the selected N‐aryl anthranilic acid scaffold can access the c‐Myb‐binding site of KIX in a consistent and chemically plausible manner, thereby justifying its progression to MD‐based stability analysis and subsequent experimental testing.

### 3.3 MM/PBSA Identifies C3 as the Strongest Predicted c‐Myb–KIX Disruptor, Whereas C1 Is Predicted to Stabilize the Protein–Protein Interface

The MM/PBSA decomposition in Table 1 shows that the four ligands do not affect the c‐Myb–CBP/p300 KIX interface in the same way, despite belonging to the same N‐aryl anthranilic acid series. Using the apo complex as the reference state $\left(\Delta G_{\mathrm{PPI}}=-59.23\pm 9.14\;\text{kcal}/\mathrm{mol}\right)$, C1 produced a more negative interaction energy $\left(-65.70\pm 9.40\;\text{kcal}/\mathrm{mol}\right)$, corresponding to a negative $\Delta \Delta G_{\mathrm{PPI}}$ of −6.47 kcal/mol, which indicates stabilization rather than disruption of the protein–protein complex. By contrast, C2, C3, and C4 all shifted $\Delta G_{\mathrm{PPI}}$ toward less favorable values than the apo reference, giving positive $\Delta \Delta G_{\mathrm{PPI}}$ values of +2.30, +4.57, and +1.40 kcal/mol, respectively, and therefore were classified as weakening/disruptive ligands. Among them, C3 showed the largest positive shift and thus emerged as the strongest predicted disruptor, followed by C2 and then C4, whereas C1 behaved in the opposite manner. The energy decomposition further suggests that these differences arise from the balance between favorable gas‐phase interactions and their compensation by solvation. C1 displayed the most favorable van der Waals contribution $\left(-67.14\pm 5.85\;\text{kcal}/\mathrm{mol}\right)$, the most favorable electrostatic term $\left(-519.60\pm 96.92\;\text{kcal}/\mathrm{mol}\right)$, and the most favorable gas‐phase interaction energy $\left(-586.74\pm 96.75\;\text{kcal}/\mathrm{mol}\right)$, which together outweighed the associated solvation penalty and led to a more stable c‐Myb–KIX complex. In contrast, although C3 also exhibited a strongly favorable electrostatic term $\left(-494.70\pm 114.43\;\text{kcal}/\mathrm{mol}\right)$, this gain was more effectively offset by solvation $\left(496.73\pm 104.87\;\text{kcal}/\mathrm{mol}\right)$, resulting in the least favorable overall $\Delta G_{\mathrm{PPI}}$ among the ligand‐bound systems $\left(-54.66\pm 10.40\;\text{kcal}/\mathrm{mol}\right)$. C2 and C4 produced intermediate effects, with more modest positive $\Delta \Delta G_{\mathrm{PPI}}$ values that still support some degree of interface weakening. Taken together, these results suggest that ligand binding to KIX does not automatically translate into disruption of the c‐Myb interaction; rather, the functional outcome depends on how binding reshapes the energetic balance at the protein–protein interface. On that basis, C3 appears to be the most promising candidate for experimental validation as a disruptor, whereas C1 is less attractive for this purpose because it is predicted to reinforce, rather than destabilize, the native complex.

**TABLE 1:** MM/PBSA energy decomposition and predicted effects of C1–C4 on the c‐Myb–CBP/p300 KIX protein–protein interaction. Binding‐energy components were calculated from the last 100 ns of the 500 ns MD trajectories and are reported as mean ± SD in kcal/mol. The apo c‐Myb–CBP/p300 KIX complex was used as the reference state. $\Delta G_{\mathrm{PPI}}$ corresponds to the total MM/PBSA interaction energy for the protein–protein complex, and $\Delta \Delta G_{\mathrm{PPI}}$ was calculated as $\Delta G_{\mathrm{PPI}}\left(\text{ligand}-\text{bound}\right)-\Delta G_{\mathrm{PPI}}\left(\mathrm{apo}\right)$. Accordingly, more positive $\Delta \Delta G_{\mathrm{PPI}}$ values indicate weakening of the c‐Myb–KIX interaction, whereas negative values indicate stabilization relative to the apo complex. $\Delta E_{\mathrm{vdw}}$, $\Delta E_{\mathrm{ele}}$, and $\Delta G_{\text{solv}}$ denote the van der Waals, electrostatic, and solvation contributions, respectively, and $\Delta G_{\mathrm{gas}}$ represents the summed gas‐phase interaction term.

Complex	Δ*E*_vdw_ (kcal/mol) mean ± SD	Δ*E*_ele_ (kcal/mol) mean ± SD	Δ*G*_solv_ (kcal/mol) mean ± SD	Δ*G*_gas_ (kcal/mol) mean ± SD	Δ*G*_PPI_ (Δtotal) mean ± SD	ΔΔ*G*_PPI_ vs. Apo (kcal/mol)	Predicted effect on PPI
c‐Myb‐CBP/p300 KIX (apo)	−62.18 ± 5.51	−397.44 ± 97.41	400.40 ± 90.28	−459.63 ± 96.80	−59.23 ± 9.14	0.00	Reference
c‐Myb‐CBP/p300 KIX‐C1	−67.14 ± 5.85	−519.60 ± 96.92	521.04 ± 89.52	−586.74 ± 96.75	−65.70 ± 9.40	−6.47	Stabilizing
c‐Myb‐CBP/p300 KIX‐C2	−59.42 ± 5.86	−446.39 ± 105.98	448.88 ± 97.62	−505.81 ± 104.88	−56.93 ± 9.77	+2.30	Weakening/Disruptive
c‐Myb‐CBP/p300 KIX‐C3	−56.70 ± 6.20	−494.70 ± 114.43	496.73 ± 104.87	−551.39 ± 112.85	−54.66 ± 10.40	+4.57	Weakening/Disruptive
c‐Myb‐CBP/p300 KIX‐C4	−60.50 ± 5.72	−427.24 ± 102.62	429.91 ± 94.75	−487.74 ± 101.72	−57.83 ± 9.53	+1.40	Weakening/Disruptive

### 3.4 PPI Disruption Metrics Identify C2 and C3 as the Clearest Interface‐Weakening Ligands, Whereas C1 Is Largely Neutral and C4 Shows a Mixed Profile

The time‐resolved PPI disruption metrics in Figure 4 and the averaged values in Table 2 show that the four N‐aryl anthranilic acid hits influence the c‐Myb–CBP/p300 KIX interface in distinct ways. Relative to the apo complex, which displayed a mean chain distance of 1.51 ± 0.07 nm, C2 and C3 produced the largest increases in interchain separation (1.56 ± 0.15 and 1.58 ± 0.08 nm, respectively), consistent with a weaker or more loosened interface. This trend was strongest for C2, which also showed the lowest number of interchain contacts at both cutoffs (197.53 ± 19.35 at < 0.45 nm and 311.68 ± 30.61 at < 0.6 nm) together with fewer hydrogen bonds (5.53 ± 2.32) than the apo system, making it the clearest contact‐based indicator of disruption. C3 likewise increased the mean chain distance, but its contact and hydrogen‐bond profile was more nuanced: although it did not reduce interface contacts as strongly as C2 (215.58 ± 27.12 and 347.32 ± 40.13 contacts; 6.73 ± 2.32 H‐bonds), it maintained the shortest ligand‐to‐interface distance (0.87 ± 0.18 nm), suggesting that this compound remains closely associated with the binding region while still promoting a rearranged interfacial geometry. In contrast, C1 behaved much more like a non‐disruptive binder, with values close to the apo state for interchain contacts (215.74 ± 17.06 and 340.64 ± 23.05) and hydrogen bonds (6.15 ± 2.05), despite a modest increase in chain distance (1.56 ± 0.08 nm); this pattern is consistent with ligand occupancy without meaningful weakening of the protein–protein interface. C4 showed the most mixed behavior because it reduced the chain distance to 1.42 ± 0.07 nm and increased interchain contacts (237.32 ± 18.41 and 400.16 ± 24.94), which argues against strong disruption even though it remained productively associated with the site (100.44 ± 13.17 ligand contacts; 1.12 ± 0.06 nm ligand distance). The reference compound Naphthol AS‐E phosphate produced the most contact‐rich and hydrogen‐bonded state overall, with the smallest chain distance (1.39 ± 0.05 nm), the highest interchain contact counts (278.66 ± 43.91 and 436.87 ± 52.50), and the largest number of hydrogen bonds (11.99 ± 4.52), indicating that under these simulation conditions it favored a compact, highly associated complex rather than a physically separated interface. Taken together, these metrics support C2 as the most straightforward contact‐based disruptor, C3 as a ligand that may weaken the interface through structural reorganization rather than simple contact loss, C1 as largely neutral, and C4 as only weakly or ambiguously disruptive.

**FIGURE 4:**
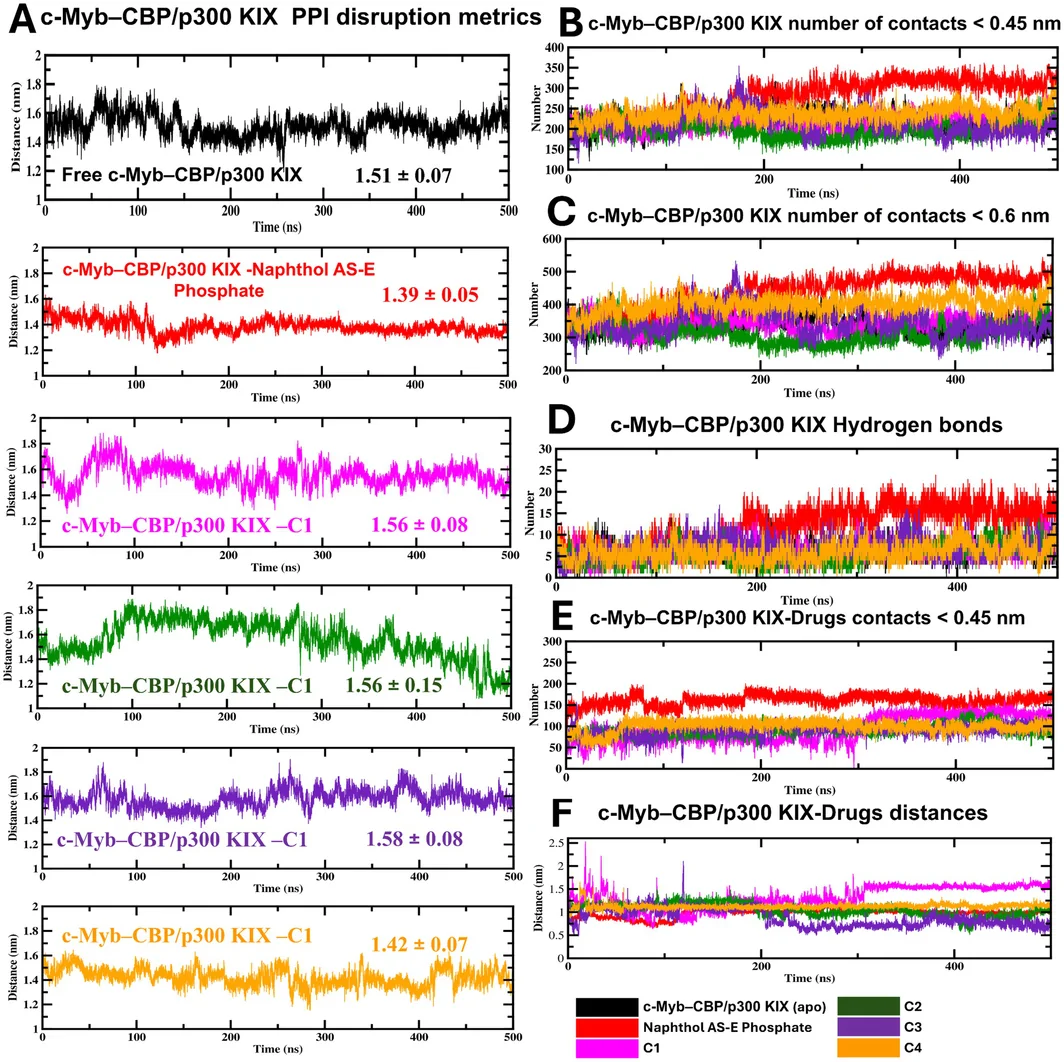
Time‐resolved metrics describing the effect of ligands on the c‐Myb–CBP/p300 KIX protein–protein interface during the 500 ns MD simulations. (A) Distance between the c‐Myb and KIX chains as a function of simulation time. (B) Number of c‐Myb–KIX intermolecular contacts using a 0.45 nm cutoff. (C) Number of c‐Myb–KIX intermolecular contacts using a 0.6 nm cutoff. (D) Number of hydrogen bonds formed between c‐Myb and KIX. (E) Number of ligand contacts with the c‐Myb–KIX complex using a 0.45 nm cutoff. (F) Distance between each ligand and the c‐Myb–KIX binding region over time. Traces are shown for the apo complex, Naphthol AS‐E phosphate, and compounds C1–C4. Mean ± SD values derived from these trajectories are summarized in Table 2.

**TABLE 2:** Average PPI disruption metrics for the c‐Myb–CBP/p300 KIX complexes obtained from the 500 ns MD simulations. Reported values are given as mean ± SD.

System	cMyb‐KIX chains distance (nm) mean ± SD	cMyb‐KIX chains contacts < 0.45 nm mean ± SD	cMyb‐KIX chains contacts < 0.6 nm mean ± SD	c‐Myb–CBP/p300 KIX H‐bonds mean ± SD	c‐Myb–CBP/p300 KIX H‐bonds pairs ≤ 0.35 nm mean ± SD	c‐Myb–CBP/p300 KIX‐drug contacts < 0.45 nm mean ± SD	c‐Myb–CBP/p300 KIX‐drug distance mean ± SD
c‐Myb–CBP/p300 KIX (apo)	1.51 ± 0.07	218.65 ± 20.22	341.46 ± 29.22	6.10 ± 1.91	2.79 ± 1.74	—	—
c‐Myb–CBP/p300 KIX‐ Naphthol AS‐E phosphate	1.39 ± 0.05	278.66 ± 43.91	436.87 ± 52.50	11.99 ± 4.52	6.78 ± 3.07	160.70 ± 13.19	0.98 ± 0.09
c‐Myb–CBP/p300 KIX‐ C1	1.56 ± 0.08	215.74 ± 17.06	340.64 ± 23.05	6.15 ± 2.05	2.54 ± 1.61	93.79 ± 30.08	1.32 ± 0.24
c‐Myb–CBP/p300 KIX‐ C2	1.56 ± 0.15	197.53 ± 19.35	311.68 ± 30.61	5.53 ± 2.32	2.73 ± 1.92	91.63 ± 12.30	1.04 ± 0.14
c‐Myb–CBP/p300 KIX‐ C3	1.58 ± 0.08	215.58 ± 27.12	347.32 ± 40.13	6.73 ± 2.32	3.46 ± 2.04	92.05 ± 12.93	0.87 ± 0.18
c‐Myb–CBP/p300 KIX‐ C4	1.42 ± 0.07	237.32 ± 18.41	400.16 ± 24.94	5.85 ± 2.10	2.40 ± 1.45	100.44 ± 13.17	1.12 ± 0.06

### 3.5 FEL Analysis Shows That C2 and C3 Promote Stable but More Reorganized Low‐Energy States, Whereas C1 and C4 Converge to More Compact Bound Conformations

The free energy landscape (FEL) analysis in Figure 5 revealed clear differences in the conformational spaces sampled by the apo and ligand‐bound c‐Myb–CBP/p300 KIX systems during the 500 ns simulations. The apo complex displayed a comparatively broad and rugged landscape with multiple low‐energy regions, indicating that the unliganded interface explores several conformational substates rather than remaining confined to a single dominant minimum. A similarly heterogeneous profile was observed for Naphthol AS‐E phosphate, which populated more than one favorable basin and therefore appears to permit substantial conformational redistribution within the complex. In contrast, the four N‐aryl anthranilic acid ligands produced more funnel‐like landscapes, consistent with convergence toward preferred bound states. Among them, C1 and C4 showed relatively compact and localized minima, suggesting that once bound they restrict the system to a narrower conformational ensemble. C2 and C3, although still characterized by dominant low‐energy basins, sampled broader regions around those minima, indicating that these compounds stabilize the complex in bound states that retain greater structural plasticity. This distinction is important because a ligand can remain stably associated with the target while still allowing, or even promoting, rearrangements at the c‐Myb–KIX interface; thus, the wider low‐energy conformational space observed for C2 and especially C3 is consistent with their more disruptive behavior in the preceding contact‐ and energy‐based analyses.

**FIGURE 5:**
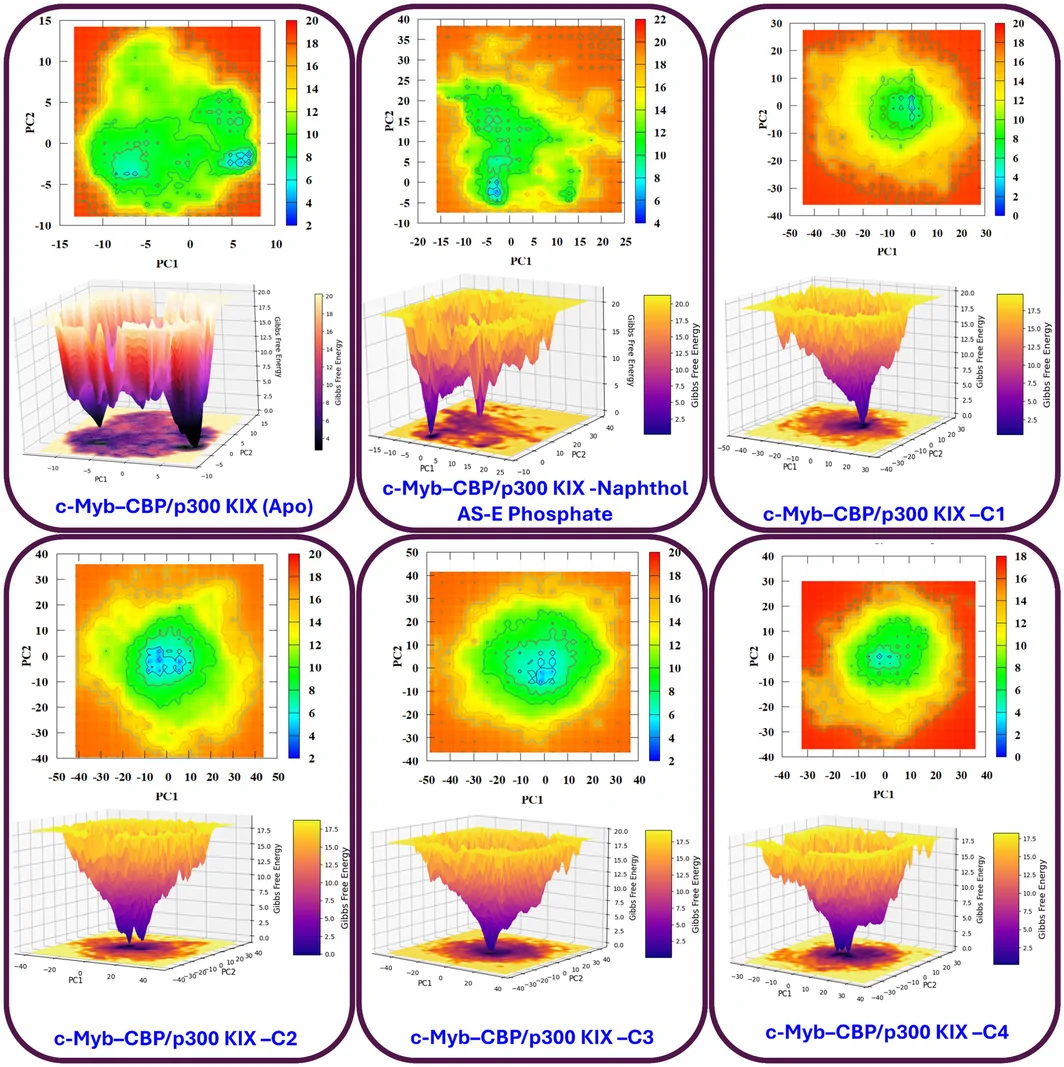
Free energy landscapes of the apo and ligand‐bound c‐Myb–CBP/p300 KIX systems derived from principal component analysis of the 500 ns MD trajectories. For each system, the upper panel shows the two‐dimensional FEL projected onto the first two principal components (PC1 and PC2), and the lower panel shows the corresponding three‐dimensional Gibbs free‐energy surface. The analyzed systems include the apo c‐Myb–CBP/p300 KIX complex, the reference ligand Naphthol AS‐E phosphate, and the four selected compounds C1–C4. Color gradients represent relative free‐energy levels, with lower‐energy basins corresponding to the most populated conformational states sampled during the simulations.

The lowest‐energy conformations extracted from the FEL minima (Figure 6) support this interpretation by showing that all four ligands remain positioned within the c‐Myb‐facing groove of KIX rather than drifting away from the target site. In these representative structures, the compounds occupy the interfacial cavity bordered by c‐Myb residues such as Arg294, Glu297, Leu298, Leu301, and Leu302 and KIX residues including Lys606, Leu607, Gln609, Ala610, Pro613, Ile657, and Gln661, confirming that the low‐energy states correspond to persistent interfacial binding modes. The reference inhibitor Naphthol AS‐E phosphate adopts a more extended and highly solvated pose, consistent with the more rugged FEL observed for this system. By comparison, C1 sits in a compact low‐energy pose that appears well packed within the groove, which agrees with its previously observed tendency to bind without clearly destabilizing the protein–protein interface. C2 and C3 also remain anchored in the same region, but their lowest‐energy structures suggest a bound arrangement that still permits interfacial reorganization, consistent with their broader FEL basins and stronger predicted disruptive effects. C4 preserves a stable groove‐bound conformation as well, but its more localized minimum suggests a more constrained bound state and therefore a weaker perturbation of the interface than that seen for C2 or C3. Taken together, the FEL results indicate that all selected compounds form stable low‐energy complexes with the target site, but that C2 and C3 are distinguished by promoting a more reorganized conformational ensemble that is more compatible with functional disruption of the c‐Myb–KIX interaction.

**FIGURE 6:**
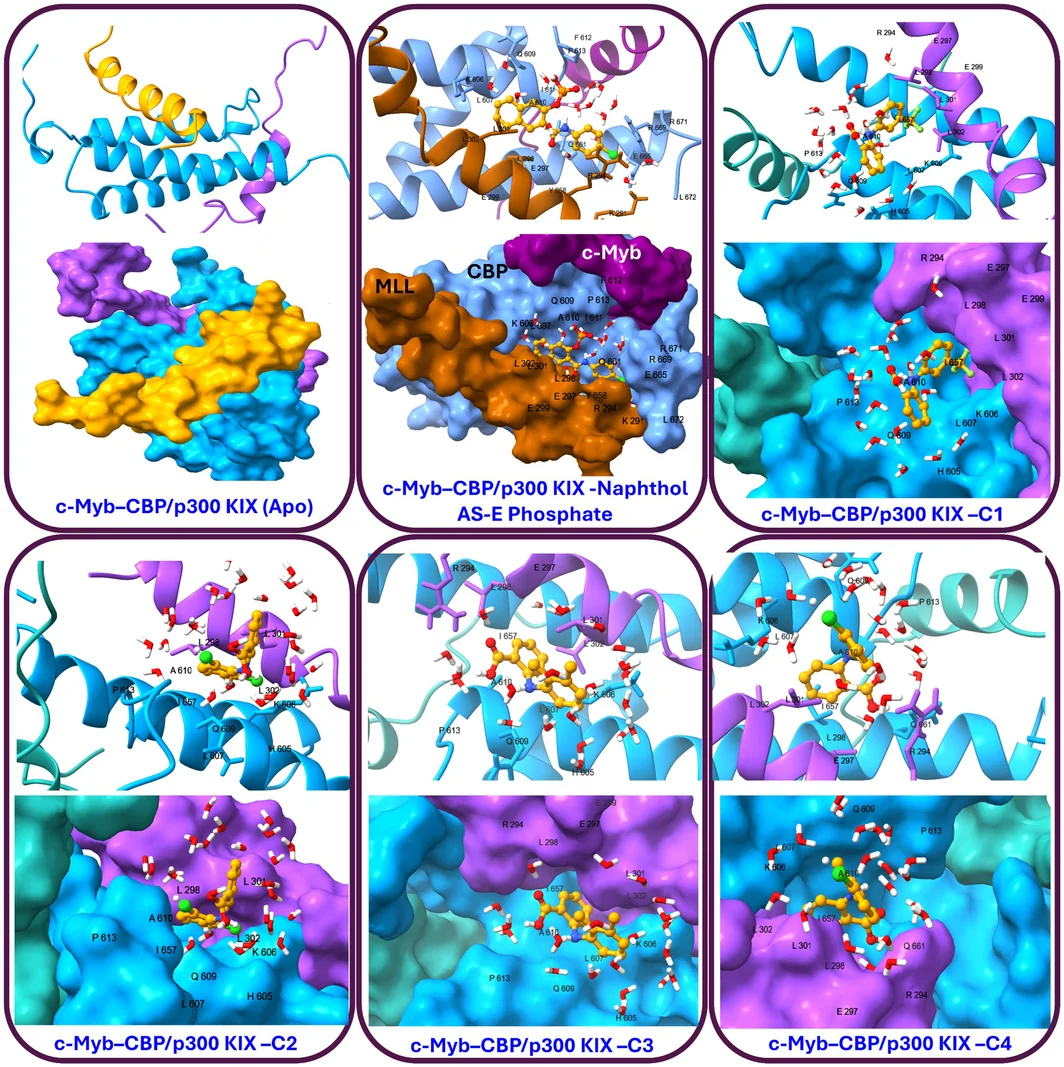
Representative lowest‐energy conformations extracted from the FEL minima of the apo and ligand‐bound c‐Myb–CBP/p300 KIX systems. For each ligand‐bound system, representative structures corresponding to the lowest‐energy basin identified in the FEL analysis are shown in cartoon and surface views to illustrate ligand placement within the c‐Myb‐facing groove of KIX. The apo complex is shown for structural comparison. The panels highlight the minimum‐energy conformations of the complex with Naphthol AS‐E phosphate and compounds C1–C4, emphasizing the persistent occupation of the interfacial pocket in the lowest‐energy states sampled during the 500 ns MD simulations.

### 3.6 MD Stability Metrics Show That C2, C3, and C4 Maintain Structurally Stable KIX‐Bound States, Whereas C1 Displays the Largest Ligand‐Associated Rearrangement

The structural stability analysis in Figure 7, Table 3 indicates that the c‐Myb–CBP/p300 KIX complexes remained globally well behaved during the 500 ns simulations, but the ligands differed in the extent to which they perturbed local versus overall structural stability. The backbone RMSD values were tightly clustered across all systems, ranging from 0.852 ± 0.133 nm for the apo complex to 1.040 ± 0.095 nm for the Naphthol AS‐E phosphate system, showing that none of the ligands caused major loss of structural integrity in the protein assembly. In contrast, the complex RMSD separated the compounds more clearly: C4 (0.550 ± 0.053 nm) and C3 (0.567 ± 0.090 nm) showed the lowest values, followed by C2 (0.648 ± 0.141 nm), indicating stable ligand‐associated conformations throughout the trajectory, whereas C1 displayed a markedly higher and more variable complex RMSD (1.290 ± 0.483 nm), consistent with the larger rearrangement seen in the time‐course trace and suggesting a less well‐converged or more mobile bound state. The radius of gyration (Rg) further supports preserved overall compactness, with only modest differences among systems; however, C4 yielded the smallest mean Rg (1.603 ± 0.027 nm) and the lowest SASA (96.28 ± 3.45 nm^2^), indicating the most compact and least solvent‐exposed complex, while C3 showed the largest Rg (1.680 ± 0.053 nm) and the highest SASA (101.34 ± 3.52 nm^2^), suggesting a slightly more expanded ensemble despite its stable RMSD behavior. C2 occupied an intermediate but favorable profile, combining low complex RMSD with modest Rg (1.651 ± 0.040 nm) and SASA (100.28 ± 3.59 nm^2^). The hydrogen‐bond traces in Figure 7E suggest that the reference ligand maintains the most persistent polar contact network, while the anthranilic acid derivatives rely on fewer and more fluctuating hydrogen bonds, implying that their stability is driven more by hydrophobic packing and shape complementarity than by sustained polar anchoring. Taken together, these results show that C2, C3, and C4 form structurally stable bound states without destabilizing the global fold of the c‐Myb–KIX complex, whereas C1 exhibits the greatest ligand‐associated mobility and therefore appears less conformationally robust than the other hits.

**FIGURE 7:**
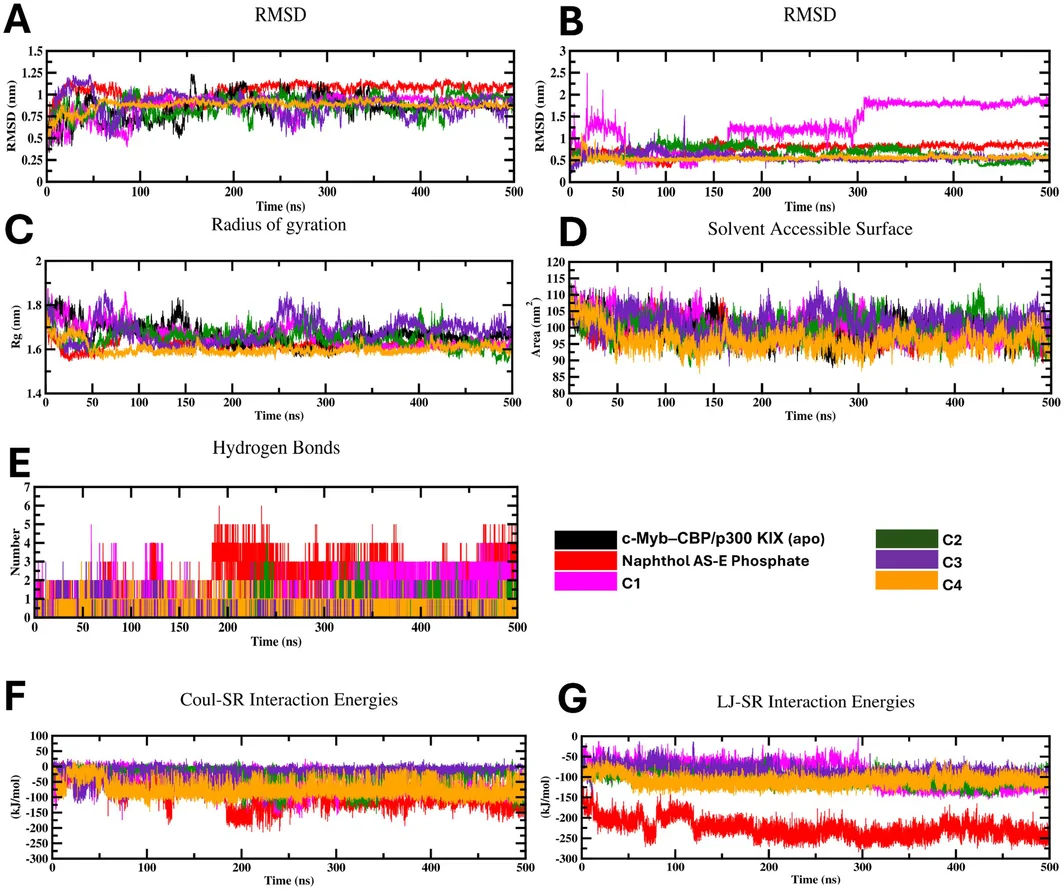
Structural stability profiles of the apo and ligand‐bound c‐Myb–CBP/p300 KIX systems during the 500 ns MD simulations. (A) Backbone RMSD of the protein complex as a function of simulation time. (B) Complex RMSD for the ligand‐bound systems, reflecting the stability of the protein–ligand assembly during the trajectory. (C) Radius of gyration (Rg) of the complex. (D) Solvent‐accessible surface area (SASA). (E) Number of protein–ligand hydrogen bonds formed over time. (F) Coul‐SR interaction energy profile. (G) LJ‐SR interaction energy profile. Traces are shown for the apo complex, Naphthol AS‐E phosphate, and compounds C1–C4. Average values for the structural stability descriptors are summarized in Table 3.

**TABLE 3:** Structural stability metrics for the apo and ligand‐bound c‐Myb–CBP/p300 KIX systems obtained from the 500 ns MD simulations. Values are reported as mean ± SD for backbone RMSD, complex RMSD, radius of gyration (Rg), and solvent‐accessible surface area (SASA).

System	Backbone RMSD (nm) mean ± SD	Complex RMSD (nm) mean ± SD	Radius of gyration, Rg (nm) mean ± SD	SASA (nm^2^) mean ± SD
c‐Myb–CBP/p300 KIX (apo)	0.852 ± 0.133	—	1.668 ± 0.046	99.40 ± 3.56
c‐Myb–CBP/p300 KIX‐ Naphthol AS‐E phosphate	1.040 ± 0.095	0.770 ± 0.111	1.624 ± 0.028	98.51 ± 3.06
c‐Myb–CBP/p300 KIX‐ C1	0.864 ± 0.138	1.290 ± 0.483	1.662 ± 0.049	100.38 ± 3.74
c‐Myb–CBP/p300 KIX‐ C2	0.867 ± 0.111	0.648 ± 0.141	1.651 ± 0.040	100.28 ± 3.59
c‐Myb–CBP/p300 KIX‐C3	0.885 ± 0.123	0.567 ± 0.090	1.680 ± 0.053	101.34 ± 3.52
c‐Myb–CBP/p300 KIX‐ C4	0.878 ± 0.061	0.550 ± 0.053	1.603 ± 0.027	96.28 ± 3.45

### 3.7 C1 and C4 Show the Most Favorable Direct Binding to the KIX‐Containing Complex, Whereas C3 Is the Weakest Binder and Disruption Potency Is Not Explained by Affinity Alone

The binding free‐energy decomposition in Table 4 shows that the four N‐aryl anthranilic acid derivatives differ substantially in their predicted affinity for the c‐Myb–CBP/p300 KIX complex and in the balance of non‐bonded forces that stabilize binding. Based on the total MM/PBSA binding energy $\left(\Delta G_{\text{total}}\right)$, C1 exhibited the most favorable overall interaction $\left(-28.18\pm 3.69\;\text{kcal}/\mathrm{mol}\right)$, followed closely by C4 $\left(-26.88\pm 2.24\;\text{kcal}/\mathrm{mol}\right)$, whereas C2 $\left(-21.10\pm 2.69\;\text{kcal}/\mathrm{mol}\right)$ and especially C3 $\left(-17.50\pm 2.31\;\text{kcal}/\mathrm{mol}\right)$ showed weaker predicted binding. The energetic breakdown indicates that these rankings arise from different interaction patterns. C4 produced the most favorable electrostatic contribution $\left(\Delta E_{\mathrm{ele}}=-25.38\pm 4.47\;\text{kcal}/\mathrm{mol}\right)$, the most favorable gas‐phase term $\left(\Delta G_{\mathrm{gas}}=-61.14\pm 5.00\;\text{kcal}/\mathrm{mol}\right)$, and the strongest short‐range non‐bonded interactions in both the Coul‐SR $\left(-74.40\pm 2.87\right)$ and LJ‐SR $\left(-109.75\pm 13.90\right)$ terms (Figure 7F,G), indicating particularly strong local contacts with the binding site; however, these gains were partly offset by the largest solvation penalty $\left(\Delta G_{\text{solv}}=34.26\pm 4.25\;\text{kcal}/\mathrm{mol}\right)$. In contrast, C1 combined favorable van der Waals (−31.57 ± 2.87) and electrostatic (16.33 ± 4.74) components with a more moderate desolvation cost (19.73 ± 3.77), yielding the most favorable net binding energy overall. C2 was characterized by strong van der Waals/Lennard‐Jones stabilization $\left(\Delta E_{\mathrm{vdw}}=-36.16\pm 2.34;\mathrm{LJ}-\mathrm{SR}=-102.42\pm 16.29\right)$ but relatively weak electrostatic support, while C3 showed the least favorable gas‐phase and short‐range interaction terms overall, consistent with its weaker direct affinity for the complex. Importantly, when these results are considered together with the earlier PPI disruption analyses, they show that stronger binding to KIX does not necessarily translate into stronger disruption of the c‐Myb–KIX interface: C1 binds most favorably yet was predicted to stabilize the protein–protein complex, whereas C3, despite having the weakest direct binding energy, emerged as the strongest predicted disruptor. This distinction suggests that the most effective ligands in this system are not simply the tightest binders, but those that most productively perturb the interfacial geometry and energetic coupling of the c‐Myb–KIX interaction.

**TABLE 4:** MM/PBSA binding free‐energy decomposition and short‐range non‐bonded interaction terms for the ligand‐bound c‐Myb–CBP/p300 KIX complexes. Energy values are reported as mean ± SD for the selected equilibrated frames from the last 100 ns of the 500 ns MD simulations.

Complex	Δ*E*_vdw_	Δ*E*_ele_	Δ*G*_solv_	Δ*G*_gas_	Δ*G*_total_	Coul‐SR	LJ‐SR
c‐Myb–CBP/p300 KIX‐ C1	−31.57 ± 2.87	−16.33 ± 4.74	19.73 ± 3.77	−47.91 ± 5.24	−28.18 ± 3.69	−49.79 ± 3.83	−91.28 ± 12.63
c‐Myb–CBP/p300 KIX‐ C2	−36.16 ± 2.34	−6.94 ± 4.50	22.00 ± 4.10	−43.10 ± 5.07	−21.10 ± 2.69	−24.23 ± 2.77	−102.42 ± 16.29
c‐Myb–CBP/p300 KIX‐ C3	−23.66 ± 2.24	−5.27 ± 4.40	11.43 ± 4.23	−28.93 ± 5.01	−17.50 ± 2.31	−19.70 ± 2.21	−85.25 ± 13.79
c‐Myb–CBP/p300 KIX‐ C4	−35.76 ± 2.24	−25.38 ± 4.47	34.26 ± 4.25	−61.14 ± 5.00	−26.88 ± 2.24	−74.40 ± 2.87	−109.75 ± 13.90

### 3.8 C3 Shows the Most Balanced Developability Profile, but All Four Hits Retain Predicted Safety and Metabolism Liabilities That Will Require Optimization

The in silico ADMET results in Table 5 indicate that all four N‐aryl anthranilic acid hits satisfy basic drug‐likeness criteria, but they also share several liabilities that would need attention in future optimization. All compounds fell within a relatively compact physicochemical range (MW 241.11–295.02 Da, TPSA 49.33 Å^2^) and showed zero Lipinski violations, supporting the suitability of this scaffold for further development. Among the series, C3 displayed the most favorable overall balance of properties, with the lowest molecular weight, the best predicted aqueous solubility (logS −4.142), the lowest lipophilicity (logD 2.949; logP 4.794), the highest predicted fraction unbound (Fu 7.427), and the largest apparent distribution volume (logVDss −0.176), suggesting comparatively better developability than the other analogues. C1 also showed a relatively acceptable profile, including no Lipinski violations, negative P‐gp substrate prediction, negative BBB penetration, negative Ames mutagenicity, negative carcinogenicity, and notably the only negative skin‐sensitization prediction in the set. In contrast, C2 appeared to be the most lipophilic analogue (logP 5.370) and the only compound predicted to cross the blood–brain barrier, while also showing very high plasma protein binding (99.27%) and acting as a predicted substrate for several metabolic enzymes (CYP1A2, CYP2C19, and CYP2C9), indicating a greater likelihood of complex disposition behavior. C4 behaved similarly to C2 in terms of high protein binding (98.99%) and low fraction unbound (0.517), although without predicted BBB penetration.

**TABLE 5:** Predicted ADMET profiles of the selected N‐aryl anthranilic acid hits (C1–C4). In silico ADMET parameters were predicted for compounds C1–C4 using ADMETlab 3.0 and are grouped into physicochemical, absorption, distribution, metabolism, excretion, and toxicity categories. Reported values include numerical property estimates as well as binary classification outputs, with the corresponding model confidence shown in parentheses.

Parameter	C1	C2	C3	C4
*Physicochemical properties*				
Lipinski's violations	0	0	0	0
Molecular weight (MW)	281.07	295.02	241.11	261.06
Partition coefficient (logP)	4.984	5.370	4.794	5.083
Distribution coefficient (logD)	3.146	3.199	2.949	3.055
Topological polar surface area (TPSA)	49.33	49.33	49.33	49.33
Aqueous solubility (logS)	−4.727	−4.492	−4.142	−4.159
*Absorption*				
PAMPA permeability	+ (0.970)	+ (0.937)	+ (0.952)	+ (0.927)
Human intestinal absorption (HIA)	− (0.009)	− (< 0.001)	− (< 0.001)	− (< 0.001)
MDCK permeability	−4.835	−4.822	−4.761	−4.812
Caco‐2 permeability	−5.033	−4.707	−4.727	−4.826
P‐gp substrate	− (0.104)	− (0.005)	+ (0.686)	− (0.196)
P‐gp inhibitor	− (0.060)	− (0.014)	− (0.002)	− (0.001)
*Distribution*				
Plasma protein binding (PPB)	91.61	99.27	92.39	98.99
Fraction unbound (Fu)	5.171	0.276	7.427	0.517
Blood–brain barrier penetration (BBB)	− (0.116)	+ (0.568)	− (0.216)	− (0.187)
Steady‐state volume of distribution (logVDss)	−0.675	−0.808	−0.176	−0.886
*Metabolism*				
CYP3A4 substrate	− (< 0.001)	− (0.320)	− (< 0.001)	− (< 0.001)
CYP3A4 inhibitor	− (< 0.001)	− (0.032)	− (0.022)	− (0.033)
CYP2D6 substrate	− (< 0.001)	− (< 0.001)	− (0.153)	− (0.005)
CYP2D6 inhibitor	− (0.073)	− (0.172)	− (0.062)	− (0.023)
CYP2C9 substrate	− (0.003)	+ (> 0.999)	+ (> 0.999)	+ (0.978)
CYP2C9 inhibitor	+ (0.971)	+ (0.998)	+ (0.928)	+ (0.980)
CYP2C19 substrate	− (< 0.001)	+ (0.794)	− (< 0.001)	− (< 0.001)
CYP2C19 inhibitor	− (0.002)	− (< 0.001)	− (< 0.001)	− (0.021)
CYP1A2 substrate	− (0.002)	+ (0.994)	− (0.004)	− (< 0.001)
CYP1A2 inhibitor	+ (> 0.999)	+ (0.932)	+ (0.995)	+ (0.997)
*Excretion*				
Plasma clearance	2.037	1.014	2.562	1.866
Predicted half‐life (t1/2)	1.437	1.651	1.111	1.151
*Toxicity*				
Respiratory toxicity	+ (0.830)	+ (0.860)	+ (0.805)	+ (0.841)
Skin sensitization	− (0.371)	+ (0.823)	+ (0.870)	+ (0.888)
Neurotoxicity	+ (0.629)	+ (0.691)	+ (0.592)	+ (0.679)
Nephrotoxicity	+ (0.873)	+ (0.804)	+ (0.795)	+ (0.865)
hERG blockade	− (0.242)	− (0.164)	− (0.131)	− (0.162)
hERG blockade at 10 μM	− (0.189)	− (0.164)	− (0.121)	− (0.151)
Genotoxicity	+ (0.988)	− (0.450)	+ (0.791)	+ (0.879)
Drug‐induced liver injury (DILI)	+ (0.910)	+ (0.986)	+ (0.951)	+ (0.978)
Carcinogenicity	− (0.030)	− (0.187)	− (0.163)	− (0.174)
Ames mutagenicity	− (0.106)	− (0.213)	− (0.344)	− (0.273)

A consistent pattern across the entire series was positive PAMPA permeability together with negative predicted HIA, suggesting that passive membrane diffusion may be feasible while intestinal absorption could still be limited by other determinants not captured by permeability alone. From a metabolism perspective, all four compounds were predicted to inhibit CYP1A2 and CYP2C9, whereas none was predicted to inhibit CYP2D6 or CYP3A4, implying a narrower but still relevant risk of metabolic drug–drug interactions. The main concern emerged from the toxicity panel: although all compounds were predicted to be negative for Ames mutagenicity, carcinogenicity, and hERG blockade, every compound was flagged as positive for DILI, nephrotoxicity, neurotoxicity, and respiratory toxicity. In addition, genotoxicity was predicted for C1, C3, and C4, whereas C2 was the only analogue that remained negative for this endpoint. Taken together, these results suggest that C3 offers the most attractive overall ADMET balance from a physicochemical and distribution standpoint, C1 may be somewhat preferable from a skin‐sensitization and transporter perspective, and C2/C4 are less attractive because of higher lipophilicity and stronger protein binding; however, the shared toxicity liabilities indicate that the current hits should be viewed as early leads.

### 3.9 MST Validates C3 as the Most Potent c‐Myb–KIX Disruptor, With C4 Matching the Reference Inhibitor and C1 Showing the Weakest Activity

The in vitro MST results in Figure 8 confirmed that the assay robustly detected both formation and disruption of the c‐Myb–CBP/p300 KIX complex. In the direct‐binding experiment (Figure 8A), titration of p300(1–672) against eGFP/hc‐Myb(241–390) gave an apparent K_d_ of 26.71 ± 0.54 μM (mean ± SD, *n* = 3), verifying that the binary interaction was measurable under the final assay conditions. In the inhibition format (Figure 8B–F), all four N‐aryl anthranilic acid hits produced concentration‐dependent changes in normalized fluorescence, consistent with disruption of the Myb–KIX interaction, but with clearly different potencies. The reference inhibitor Naphthol AS‐E phosphate showed an IC_50_ of 28.8 ± 3.1 μM (Figure 8B), providing a benchmark for assay responsiveness. Among the new compounds, C3 was the most potent with an IC_50_ of 16.5 ± 0.8 μM (Figure 8E), indicating stronger inhibitory activity than the reference compound and establishing it as the lead molecule of the series. C4 was the next most active analogue, with an IC_50_ of 27.2 ± 2.7 μM (Figure 8F), essentially matching the activity of Naphthol AS‐E phosphate. C2 showed moderate inhibition with an IC_50_ of 54.7 ± 22.1 μM (Figure 8D), whereas C1 was the least active compound, with an IC_50_ of 99.6 ± 20.5 μM (Figure 8C). This experimental ranking, C3 > C4 ≈ Naphthol AS‐E phosphate > C2 > C1, is in good overall agreement with the computational analyses, particularly the PPI disruption and MM/PBSA results, which had already identified C3 as the strongest predicted disruptor and C1 as the least desirable candidate because of its stabilizing or weakly perturbing behavior. The strong activity of C3 is especially notable because its direct binding energy was not the most favorable among the series, reinforcing the conclusion that effective disruption of the c‐Myb–KIX interface depends more on how a ligand perturbs the protein–protein interaction than on binding affinity alone. Likewise, the comparable performance of C4 and the reference inhibitor suggests that the N‐aryl anthranilic acid scaffold can reproduce biologically relevant functional inhibition even when its computational profile appears more mixed. Overall, the MST data provide direct experimental support for the computational workflow and identify C3, followed by C4, as the most promising leads for further optimization as c‐Myb–KIX interaction disruptors.

**FIGURE 8:**
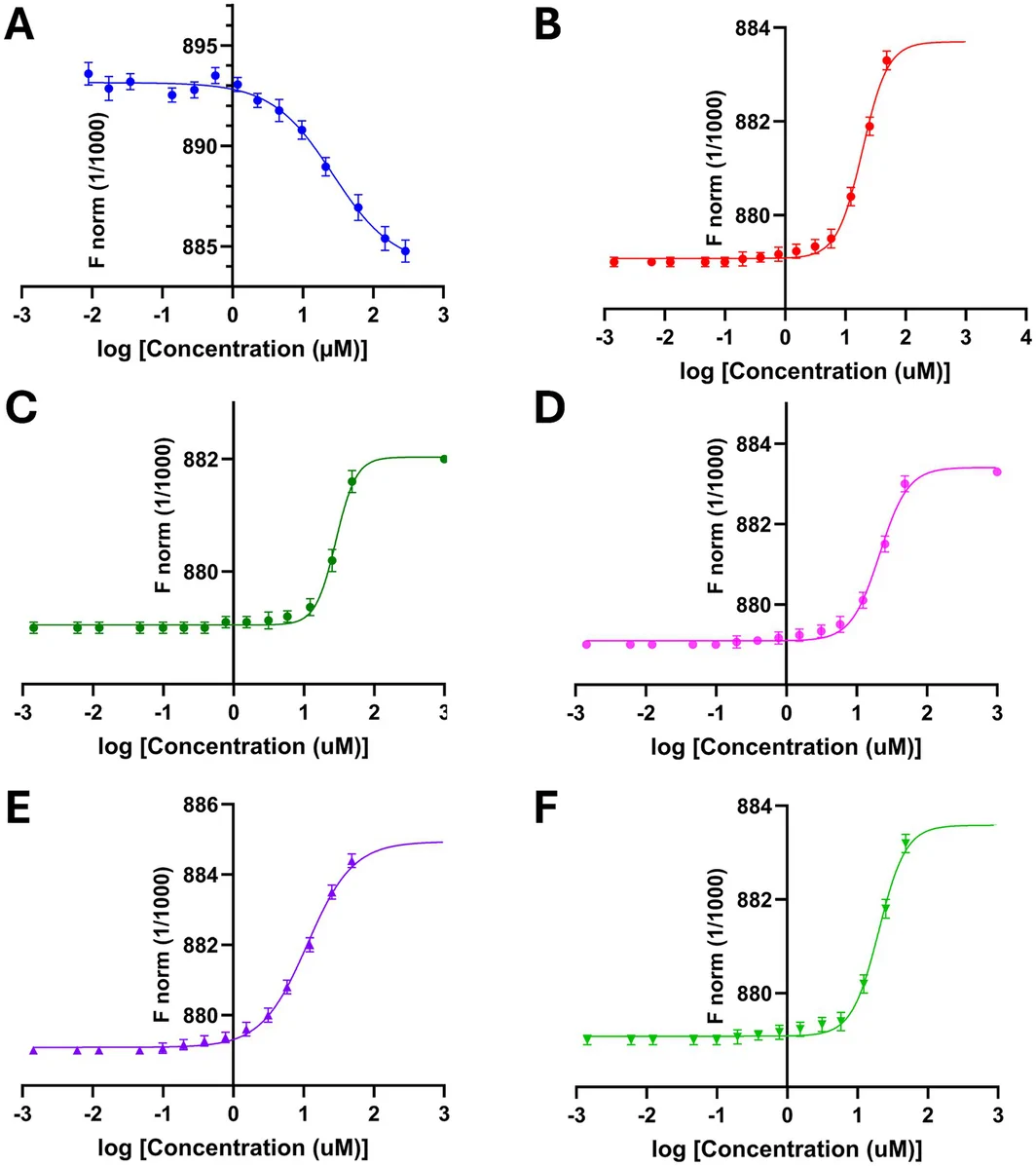
Microscale thermophoresis validation of c‐Myb–CBP/p300 KIX binding and inhibition by the selected compounds. (A) Direct‐binding MST titration of p300(1–672) against eGFP/hc‐Myb(241–390), yielding an apparent *K*_*d*_ of 26.71 ± 0.54 μM (mean ± SD, *n* = 3). (B–F) Concentration‐dependent inhibition curves obtained from the Myb–KIX MST competition assay for Naphthol AS‐E phosphate (B), C1 (C), C2 (D), C3 (E), and C4 (F). Normalized fluorescence (*F*_norm_) is plotted against the logarithm of compound concentration, and curves were fitted by nonlinear regression using a variable‐slope sigmoidal model.

A preliminary structure–activity relationship can be inferred because C1–C4 contain the same anthranilic acid headgroup and differ primarily in the substitution pattern of the N‐aryl ring. The shared carboxylic acid and diarylamine functionalities are expected to preserve the principal polar anchoring and aromatic recognition features, whereas the N‐aryl substituents modulate hydrophobic complementarity, steric demand, torsional preference, and conformational adaptability within the shallow KIX groove. C3, the most active compound, contains a compact 2,3‐dimethyl substitution pattern. The ortho‐methyl group may favor a non‐coplanar arrangement of the two aromatic rings and thereby partially preorganize the scaffold for binding, while the adjacent methyl group provides additional hydrophobic surface without imposing the steric bulk associated with chlorine or trifluoromethyl substituents. This balance may permit both stable groove occupation and sufficient local adaptability to perturb the c‐Myb–KIX interface. Consistent with this interpretation, C4, which retains the ortho‐methyl group but replaces the second methyl with chlorine, showed slightly lower but comparable activity. By contrast, the two ortho chlorine substituents of C2 generate a more sterically congested and conformationally restricted aryl environment that may hinder optimal accommodation, whereas C1 lacks an ortho substituent and carries a bulky meta‐trifluoromethyl group, consistent with its greater ligand displacement and lower experimental activity. Overall, the initial SAR suggests that compact ortho substitution, particularly an ortho‐methyl group combined with moderate hydrophobic substitution, may be advantageous for this scaffold. Nevertheless, because only four closely related compounds were examined, this interpretation should be considered preliminary and should be tested through systematic modification of the ortho and meta positions in future analogue series.

The synthetic accessibility of the N‐aryl anthranilic acid scaffold supports further medicinal‐chemistry optimization. Future studies could systematically vary ortho, meta, and para substituents to balance steric fit, hydrophobicity, and electronic effects, while also exploring benzoic‐acid‐ring substitutions, carboxylate bioisosteres, and conformationally restricted analogues. Extension toward adjacent shallow pockets may further improve potency and selectivity. These modifications should be optimized together with solubility, permeability, metabolic stability, and selectivity for the c‐Myb‐binding surface of KIX. Future studies also should determine whether the biochemical inhibition observed by MST translates into activity in MYB‐dependent AML models. Priority experiments include evaluating C3 in AML cell lines, assessing cell viability and proliferation, measuring changes in MYB‐responsive gene expression, and confirming intracellular engagement of the c‐Myb–KIX axis. These studies will establish the cellular relevance, selectivity, and therapeutic potential of the N‐aryl anthranilic acid scaffold.

## 4 Conclusion

In this study, we established an integrated in silico–in vitro workflow to discover new N‐aryl anthranilic acid inhibitors of the c‐Myb–CBP/p300 KIX protein–protein interaction. Starting from a scaffold‐focused library of 3656 compounds, a stepwise screening strategy combining drug‐likeness filtering, docking, MD refinement, contact analysis, MM/PBSA evaluation, and ADMET prediction reduced the search space to four prioritized candidates (C1–C4). Docking and long‐timescale MD simulations showed that these compounds consistently targeted the α1/α3 c‐Myb‐binding groove of KIX, but the subsequent energetic and dynamic analyses made clear that binding alone was not sufficient to predict functional disruption. Instead, the most informative descriptors were those that captured how ligand binding altered the energetic and conformational balance of the c‐Myb–KIX interface. Within this framework, C3 emerged as the strongest predicted disruptor, C2 showed weaker but detectable disruptive behavior, C4 displayed a mixed computational profile, and C1 was predicted to be the least favorable candidate because of its stabilizing tendency toward the protein–protein complex. These computational predictions were supported by microscale thermophoresis, which experimentally confirmed disruption of the Myb–KIX interaction by the selected compounds. Among the tested molecules, C3 showed the highest activity with an IC₅₀ of 16.5 ± 0.8 μM, outperforming the reference inhibitor Naphthol AS‐E phosphate (28.8 ± 3.1 μM), while C4 exhibited comparable potency (27.2 ± 2.7 μM). In contrast, C2 was only moderately active and C1 was the weakest inhibitor, in good agreement with the overall computational ranking. Together, these findings identify C3 as the most promising lead from this series and demonstrate that N‐aryl anthranilic acids provide a tractable chemical scaffold for targeting the c‐Myb–CBP/p300 KIX interface. More broadly, the study shows that combining structure‐based screening with dynamic and energetic analyses can effectively prioritize functional PPI disruptors, providing a clear basis for future medicinal‐chemistry optimization of this scaffold toward improved potency, selectivity, and developability.

## Author Contributions

**Haifa E. Alfassam:** data curation, formal analysis, visualization, project administration, supervision. **Emadeldin M. Kamel:** data curation, conceptualization, formal analysis, visualization, investigation, writing – review and editing, writing – original draft, supervision. **Sarah I. Othman:** conceptualization, data curation, investigation, validation, software. **Hanan M. Alharbi:** investigation, supervision, project administration. **Hassan A. Rudayni:** visualization, formal analysis, data curation. **Ahmed A. Allam:** data curation, conceptualization, funding acquisition, resources. **Al Mokhtar Lamsabhi:** writing – original draft, writing – review and editing, data curation, formal analysis, supervision, methodology, investigation.

## Funding

The authors extend their appreciation to the Deanship of Scientific Research and Libraries in Princess Nourah bint Abdulrahman University for funding this research work through the Research Group project, Grant (RG‐1445‐0050).

## Ethics Statement

The authors have nothing to report.

## Consent

The authors have nothing to report.

## Conflicts of Interest

The authors declare no conflicts of interest.

## Supporting information


**File S1:** Excel spreadsheet containing the library of compounds used in this study.

## Data Availability

The authors have nothing to report.

## References

[cbdd70415-bib-0001] Alqhtani, H. A. , S. I. Othman , F. F. A. Alkhayl , N. G. Altoom , A. M. Lamsabhi , and E. M. Kamel . 2025. “Inhibitory Mechanisms of β‐Glucuronidase by *Hibiscus syriacus* Phenolics: Integrating Computational and Experimental Approaches.” ChemistrySelect 10, no. 1: e202402984. 10.1002/slct.202402984.

[cbdd70415-bib-0002] Alwaili, M. A. , F. F. A. Alkhayl , H. A. Rudayni , A. A. Allam , A. M. Lamsabhi , and E. M. Kamel . 2025. “Mechanistic Insights Into β‐Glucuronidase Inhibition by Isoprenylated Flavonoids From Centaurea Scoparia: Bridging Experimental and Computational Approaches.” Journal of Molecular Structure 1322: 140354. 10.1016/j.molstruc.2024.140354.

[cbdd70415-bib-0003] Arkin, M. R. , Y. Tang , and J. A. Wells . 2014. “Small‐Molecule Inhibitors of Protein‐Protein Interactions: Progressing Toward the Reality.” Chemistry & Biology 21, no. 9: 1102–1114. 10.1016/j.chembiol.2014.09.001. PMC4179228 25237857

[cbdd70415-bib-0004] Baell, J. B. , and G. A. Holloway . 2010. “New Substructure Filters for Removal of Pan Assay Interference Compounds (PAINS) From Screening Libraries and for Their Exclusion in Bioassays.” Journal of Medicinal Chemistry 53, no. 7: 2719–2740. 10.1021/jm901137j. 20131845

[cbdd70415-bib-0005] Bussi, G. , D. Donadio , and M. Parrinello . 2007. “Canonical Sampling Through Velocity Rescaling.” Journal of Chemical Physics 126, no. 1: 014101. 10.1063/1.2408420. 17212484

[cbdd70415-bib-0006] De Guzman, R. N. , N. K. Goto , H. J. Dyson , and P. E. Wright . 2006. “Structural Basis for Cooperative Transcription Factor Binding to the CBP Coactivator.” Journal of Molecular Biology 355, no. 5: 1005–1013. 10.1016/j.jmb.2005.09.059. 16253272

[cbdd70415-bib-0007] Frisch, M. J. , G. W. Trucks , H. B. Schlegel , et al. 2016. “Gaussian 16 Rev. C.01.” Wallingford, CT.

[cbdd70415-bib-0008] Fu, L. , S. Shi , J. Yi , et al. 2024. “ADMETlab 3.0: An Updated Comprehensive Online ADMET Prediction Platform Enhanced With Broader Coverage, Improved Performance, API Functionality and Decision Support.” Nucleic Acids Research 52, no. W1: W422–W431. 10.1093/nar/gkae236. PMC11223840 38572755

[cbdd70415-bib-0009] Genheden, S. , and U. Ryde . 2015. “The MM/PBSA and MM/GBSA Methods to Estimate Ligand‐Binding Affinities.” Expert Opinion on Drug Discovery 10, no. 5: 449–461. 10.1517/17460441.2015.1032936. PMC4487606 25835573

[cbdd70415-bib-0010] Hess, B. , H. Bekker , H. J. C. Berendsen , and J. G. E. M. Fraaije . 1997. “LINCS: A Linear Constraint Solver for Molecular Simulations.” Journal of Computational Chemistry 18, no. 12: 1463–1472. 10.1002/(SICI)1096-987X(199709)18:12<1463::AID-JCC4>3.0.CO;2-H.

[cbdd70415-bib-0011] Huang, J. , S. Rauscher , G. Nawrocki , et al. 2017. “CHARMM36m: An Improved Force Field for Folded and Intrinsically Disordered Proteins.” Nature Methods 14, no. 1: 71–73. 10.1038/nmeth.4067. PMC5199616 27819658

[cbdd70415-bib-0012] Joy, S. T. , M. J. Henley , S. N. De Salle , et al. 2021. “A Dual‐Site Inhibitor of CBP/p300 KIX Is a Selective and Effective Modulator of Myb.” Journal of the American Chemical Society 143, no. 37: 15056–15062. 10.1021/jacs.1c04432. PMC13334770 34491719

[cbdd70415-bib-0013] Kamel, E. M. , S. M. Khadrawy , A. A. Allam , et al. 2026. “Discovery of [1,2,4]Triazolo[1,5‐a]Pyrimidine‐Based Disruptors of the Skp2–Cks1 Protein–Protein Interaction Through Integrated Computational and Experimental Analysis.” Biochemical and Biophysical Research Communications 818: 153783. 10.1016/j.bbrc.2026.153783. 42019241

[cbdd70415-bib-0015] Ledsaak, M. , M. Bengtsen , A.‐K. Molværsmyr , et al. 2016. “PIAS1 Binds p300 and Behaves as a Coactivator or Corepressor of the Transcription Factor c‐Myb Dependent on SUMO‐Status.” Biochimica et Biophysica Acta (BBA) ‐ Gene Regulatory Mechanisms 1859, no. 5: 705–718. 10.1016/j.bbagrm.2016.03.011. 27032383

[cbdd70415-bib-0016] Lee, C. , W. Yang , and R. G. Parr . 1988. “Development of the Colle‐Salvetti Correlation‐Energy Formula Into a Functional of the Electron Density.” Physical Review B 37, no. 2: 785–789. 10.1103/PhysRevB.37.785. 9944570

[cbdd70415-bib-0017] Lindahl, E. , B. Hess , and D. van der Spoel . 2001. “GROMACS 3.0: A Package for Molecular Simulation and Trajectory Analysis.” Molecular Modeling Annual 7, no. 8: 306–317. 10.1007/s008940100045.

[cbdd70415-bib-0018] Morris, G. M. , R. Huey , W. Lindstrom , et al. 2009. “AutoDock4 and AutoDockTools4: Automated Docking With Selective Receptor Flexibility.” Journal of Computational Chemistry 30, no. 16: 2785–2791. 10.1002/jcc.21256. PMC2760638 19399780

[cbdd70415-bib-0019] Pattabiraman, D. R. , C. McGirr , K. Shakhbazov , et al. 2014. “Interaction of c‐Myb With p300 Is Required for the Induction of Acute Myeloid Leukemia (AML) by Human AML Oncogenes.” Blood 123, no. 17: 2682–2690. 10.1182/blood-2012-02-413187. 24596419

[cbdd70415-bib-0020] Ramaswamy, K. , L. Forbes , G. Minuesa , et al. 2018. “Peptidomimetic Blockade of MYB in Acute Myeloid Leukemia.” Nature Communications 9, no. 1: 110. 10.1038/s41467-017-02618-6. PMC5760651 29317678

[cbdd70415-bib-0021] Schmidt, T. J. , and K.‐H. Klempnauer . 2022. “Natural Products With Antitumor Potential Targeting the MYB‐C/EBPβ‐p300 Transcription Module.” Molecules 27, no. 7: 2077. 10.3390/molecules27072077. PMC9000602 35408476

[cbdd70415-bib-0022] Smith, M. C. , and J. E. Gestwicki . 2012. “Features of Protein‐Protein Interactions That Translate Into Potent Inhibitors: Topology, Surface Area and Affinity.” Expert Reviews in Molecular Medicine 14: e16. 10.1017/erm.2012.10. PMC3591511 22831787

[cbdd70415-bib-0023] Thakur, J. K. , A. Yadav , and G. Yadav . 2014. “Molecular Recognition by the KIX Domain and Its Role in Gene Regulation.” Nucleic Acids Research 42, no. 4: 2112–2125. 10.1093/nar/gkt1147. PMC3936767 24253305

[cbdd70415-bib-0024] Trott, O. , and A. J. Olson . 2010. “AutoDock Vina: Improving the Speed and Accuracy of Docking With a New Scoring Function, Efficient Optimization, and Multithreading.” Journal of Computational Chemistry 31, no. 2: 455–461. 10.1002/jcc.21334. PMC3041641 19499576

[cbdd70415-bib-0025] Uttarkar, S. , S. Dukare , B. Bopp , M. Goblirsch , J. Jose , and K. H. Klempnauer . 2015. “Naphthol AS‐E Phosphate Inhibits the Activity of the Transcription Factor Myb by Blocking the Interaction With the KIX Domain of the Coactivator p300.” Molecular Cancer Therapeutics 14, no. 6: 1276–1285. 10.1158/1535-7163.Mct-14-0662. 25740244

[cbdd70415-bib-0026] Uttarkar, S. , J. Frampton , and K. H. Klempnauer . 2017. “Targeting the Transcription Factor Myb by Small‐Molecule Inhibitors.” Experimental Hematology 47: 31–35. 10.1016/j.exphem.2016.12.003. 28017646

[cbdd70415-bib-0027] Uttarkar, S. , T. Piontek , S. Dukare , et al. 2016. “Small‐Molecule Disruption of the Myb/p300 Cooperation Targets Acute Myeloid Leukemia Cells.” Molecular Cancer Therapeutics 15, no. 12: 2905–2915. 10.1158/1535-7163.Mct-16-0185. 27707899

[cbdd70415-bib-0028] Valdés‐Tresanco, M. S. , M. E. Valdés‐Tresanco , P. A. Valiente , and E. Moreno . 2021. “gmx_MMPBSA: A New Tool to Perform End‐State Free Energy Calculations With GROMACS.” Journal of Chemical Theory and Computation 17, no. 10: 6281–6291. 10.1021/acs.jctc.1c00645. 34586825

[cbdd70415-bib-0029] Van Der Spoel, D. , E. Lindahl , B. Hess , G. Groenhof , A. E. Mark , and H. J. C. Berendsen . 2005. “GROMACS: Fast, Flexible, and Free.” Journal of Computational Chemistry 26, no. 16: 1701–1718. 10.1002/jcc.20291. 16211538

[cbdd70415-bib-0030] Vanommeslaeghe, K. , and A. D. MacKerell Jr. 2012. “Automation of the CHARMM General Force Field (CGenFF) I: Bond Perception and Atom Typing.” Journal of Chemical Information and Modeling 52, no. 12: 3144–3154. 10.1021/ci300363c. PMC3528824 23146088

[cbdd70415-bib-0031] Yusenko, M. V. , A. Trentmann , D. A. Casolari , et al. 2021. “C/EBPβ Is a MYB‐ and p300‐Cooperating Pro‐Leukemogenic Factor and Promising Drug Target in Acute Myeloid Leukemia.” Oncogene 40, no. 29: 4746–4758. 10.1038/s41388-021-01800-x. PMC8298201 33958723

[cbdd70415-bib-0032] Zor, T. , R. N. De Guzman , H. J. Dyson , and P. E. Wright . 2004. “Solution Structure of the KIX Domain of CBP Bound to the Transactivation Domain of c‐Myb.” Journal of Molecular Biology 337, no. 3: 521–534. 10.1016/j.jmb.2004.01.038. 15019774

